# Four Novel Cellulose Synthase (CESA) Genes from *Birch* (*Betula platyphylla* Suk.) Involved in Primary and Secondary Cell Wall Biosynthesis

**DOI:** 10.3390/ijms131012195

**Published:** 2012-09-25

**Authors:** Xuemei Liu, Qiuyu Wang, Pengfei Chen, Funan Song, Minxiao Guan, Lihua Jin, Yucheng Wang, Chuanping Yang

**Affiliations:** 1 Northeast Forestry University, 26 Hexing Road Xiangfang District, Harbin 150040, China; E-Mails: liuxuemei@nefu.edu.cn (X.L.); wqyll@sina.com (Q.W.); cpfnefu1234@sohu.com (P.C.); sfn@nefu.edu.cn (F.S.); gmx.06@163.com (M.G.); lhjin2000@hotmail.com (L.J.); 2 State Key Laboratory of Forest Genetics and Tree Breeding, Northeast Forestry University, 26 Hexing Road, Harbin 150040, China

**Keywords:** birch, CESA, primary cell wall, secondary cell wall, transcript, wood, cellulose, gene expression

## Abstract

Cellulose synthase (CESA), which is an essential catalyst for the generation of plant cell wall biomass, is mainly encoded by the *CesA* gene family that contains ten or more members. In this study; four full-length cDNAs encoding CESA were isolated from *Betula platyphylla* Suk., which is an important timber species, using RT-PCR combined with the RACE method and were named as *BplCesA3*, *−4*, *−7* and *−8*. These deduced CESAs contained the same typical domains and regions as their *Arabidopsis* homologs. The cDNA lengths differed among these four genes, as did the locations of the various protein domains inferred from the deduced amino acid sequences, which shared amino acid sequence identities ranging from only 63.8% to 70.5%. Real-time RT-PCR showed that all four *BplCesAs* were expressed at different levels in diverse tissues. Results indicated that BplCESA8 might be involved in secondary cell wall biosynthesis and floral development. BplCESA3 appeared in a unique expression pattern and was possibly involved in primary cell wall biosynthesis and seed development; it might also be related to the homogalacturonan synthesis. BplCESA7 and BplCESA4 may be related to the formation of a cellulose synthase complex and participate mainly in secondary cell wall biosynthesis. The extremely low expression abundance of the four BplCESAs in mature pollen suggested very little involvement of them in mature pollen formation in *Betula*. The distinct expression pattern of the four *BplCesAs* suggested they might participate in developments of various tissues and that they are possibly controlled by distinct mechanisms in *Betula.*

## 1. Introduction

Cellulose, a key structural component of the plant cell wall, is the most abundant biopolymer in the world. Cellulose is a homopolymer consisting of β-1,4-glucan chains that are synthesized at the plasma membrane by membrane-localized “rosette” complexes [1], as visualized by freeze-fracture electron microscopy [2–5]. To date, cellulose synthase (CESA) has been localized in these cellulose-synthesizing complexes. CESA plays a central role in plant cell wall biomass formation [1,6]. Each plant synthesizes a number of different cellulose synthases and each cellulose-synthesizing complex contains at least three non-redundant cellulose synthase isoforms. Both genetic and biochemical evidence suggest that different CESA isoforms interact to form a functional cellulose synthase enzyme complex [7]. CESA uses UDP-glucose as a substrate and polymerizes glucose residues in a single-step reaction [2]. *CesA* genes are part of a *CesA*/*CSL* superfamily, and the proteins they encode contain the same domains and regions as CESA proteins of coniferous gymnosperms [8], angiosperms and molds [9].

Most higher plants express two contrasting groups of apparently co-regulated *CesA*s involved in either primary or secondary cell wall biosynthesis. The *Arabidopsis* genome encodes 10 *CesA* genes belonging to six classes known to participate in cellulose microfibril biosynthesis [10]. Expression of at least three different gene products, AtCESA1, AtCESA3 and one of the CESA6-related CESAs (AtCESA2, AtCESA5, AtCESA6 or AtCESA9), are required for primary cell wall formation. AtCESA4, AtCESA7 and AtCESA8 are required for the development of the thick secondary wall [11–13]. In rice, 45 sequences that significantly matched the *CesA*/*CSL* superfamily were revealed by searching the TIGR database, of which 11 were predicted as *OsCesA. OsCesA1*, *−3*, and *−8* showed high co-expression in the tissues of the primary cell wall, whereas *OsCesA4*, *−7*, and *−9* were co-expressed in the secondary cell wall tissues [14]. In corn, three of the 12 *CesA* genes are involved in cellulose synthesis during secondary wall formation [2,15]. Trees are perennial species that accumulate massive amounts of secondary xylem (*i.e.*, wood) [16]. Within the *CesA* family of some tree species, complexes exist that are engaged in the deposition of cellulose during primary cell wall synthesis and secondary cell wall formation similar to *Arabidopsis* and rice. In aspen (*Populus tremuloides* Mickx.), *PtdCesA1* [17], *PtdCesA2* [18] and *PtdCesA3* [19] are associated with secondary cell wall development. *PtdCesA4*, *PtdCesA5*, *PtdCesA6* and *PtdCesA7* are involved in primary cell wall development in aspen trees [20–22]. Here, it should be noted that all *CesAs* in *Populus* were renamed according to the nomenclature for the cellulose synthase genes in *P. trichocarpa* proposed by Kumar *et al*. in 2009 [23]. Microarray profiling in *P. trichocarpa* has demonstrated that *PtiCesA3-C*, *4*, *6-C*, *7-A*, *8-A* and *8-B* were clearly xylem-specific, while remaining *PtiCesA* having detectable transcript molecules did not exhibit clear tissue specificity. *PtiCesA4*, *PtiCesA7-A*, -*B* and *PtiCesA8-A*, *-B* are the homologs of *Arabidopsis AtCesA4*, *AtCesA7* and *AtCesA8*, respectively [24]. The three *AtCesA* genes are required for the biosynthesis of cellulose in the secondary cell walls [11–13]. Gene expression analysis by QRT-PCR in *Eucalyptus*, another tree species of great commercial interest, indicated that transcripts of *EgCesA1* to *3* were related to development of secondary cell walls [25]. Expression of *EgCesA4* and *EgCesA5* was related to primary wall synthesis, whereas *EgCesA6* was weakly expressed in all tissues [25]. Furthermore, eight *CesA* genes were identified in the coniferous gymnosperm *Pinus radiata* [8]. Nairn and Haselkorn [26] described three secondary cell wall-associated *CesA*s from loblolly pine gymnosperm (*Pinus taeda* L.), while recent research reported 10 *CesAs* in *Pinus taeda* although there was little evidence for their involvement in cell wall biosynthesis [27].

*Betula platyphylla* Suk., a fibrous, broadleaf commercial tree species widely distributed in the northeast of China, has many applications in architecture, furniture and paper production. However, some important economic characteristics of many tree species, such as the quality and properties of the wood, are controlled by multiple genes with undetermined genetic mechanisms. Thus, understanding the mechanism of cellulose formation would enable the improvement of fiber characteristics and the creation of new birch cultivars using biotechnology. We isolated four members of the *CesA* family from *B. platyphylla* and identified the structural elements of their deduced protein sequences. The tissue-specific expression patterns of these genes were compared at different developmental stages in *Betula*.

## 2. Results

### 2.1. Cloning the *CesA* Genes from *B. platyphylla*

Four full-length candidate *CesA* cDNA sequences were isolated from leaves and stems of *B. platyphylla*. A search for conserved domains or functional motifs in the CDD revealed that the four encoded proteins possessed two separate conserved domains: a glycosyltransferase domain (*E* value = 2 × 10^−11^) and a cellulose synthase domain, which are typical of CESAs (*E* value < 1.0 × 10^−180^) [2,4]. Therefore, the four genes were confirmed as *CesA*s and designated *BplCesA3*, *−4*, *−7* and *−8*, using the three-letter prefix nomenclature (*Bpl*) for cellulose synthase genes reported in *Populus* [23].

### 2.2. Structure and Properties of BplCESAs of *B. platyphylla*

**Figure 1:**
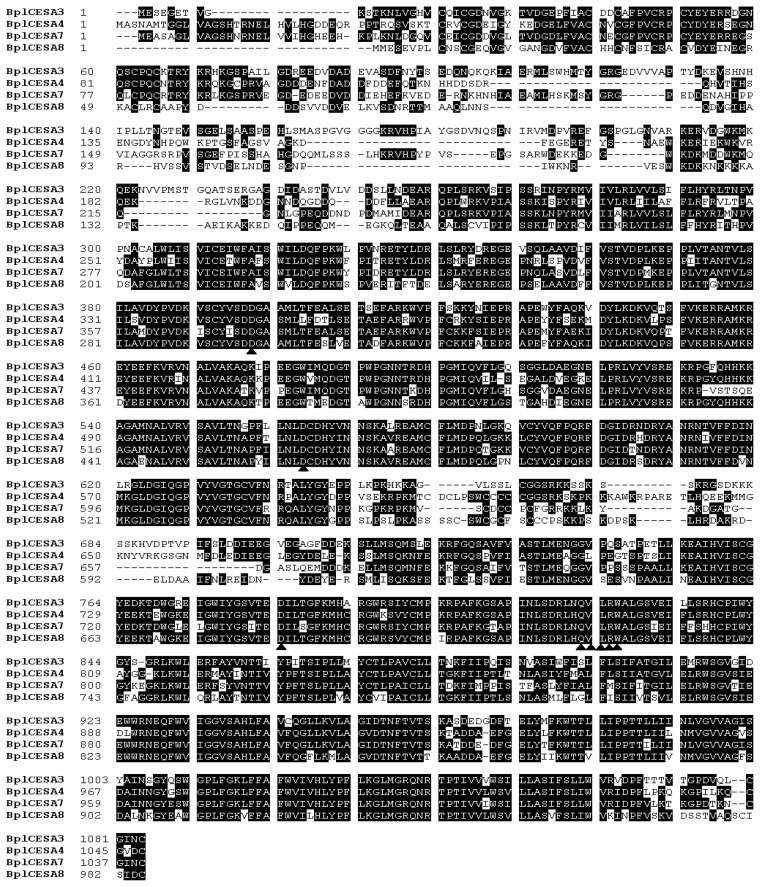
Multiple sequence alignment and main domains for the deduced amino acid sequences of four CESA proteins. Shown are the conserved processive glycosyltransferase motif (D, D, D, QVLRW, black triangles), zinc finger motif, hypervariable regions (HVRI and HVRII) and the plant conserved region (CRP). Black shading indicates amino acid identities.

**Figure 2:**
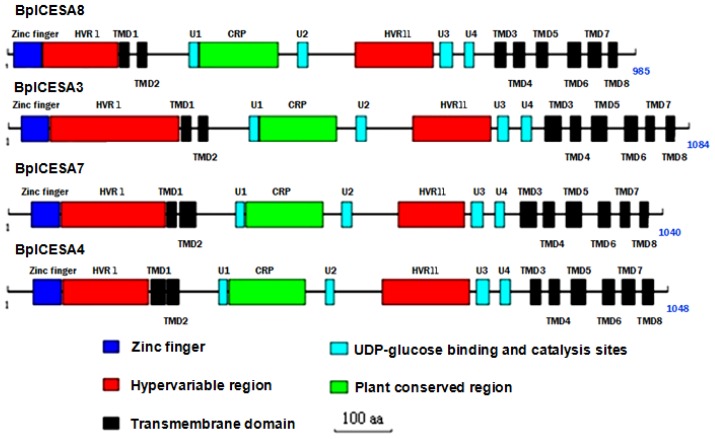
Diagrammatic representation of the four deduced CESA amino acid sequences of *Betula*. Blue, zinc finger region; black, eight transmembrane domains; green, CRP domain; red, HVRI and HVRII/CSR domains; light blue, motifs necessary for glycosyl transferase processivity [D, D, D, QXXRW (U1–U4)].

The four full-length *CesA*s ranged from 3255 to 3968 bp in length, containing ORF lengths ranging from 2958 to 3255 bp, encoding 985 to 1084 amino acids (Table 1). The four putative BplCESAs differed at residues 11 to 33 of the *N* terminus and 16 residues at the C terminus (Figure 1) and shared only 63.8% to 70.5% identity (Table 1), which was similar to that shared by the seven aspen CESAs (64%–76%) [18,22]. However, the putative BplCESAs shared the highest identity (85%–98%) with orthologs in other plants. Importantly, the four BplCESAs contained all the typical motifs of plant cellulose synthases described by Joshi [19], including the plant conserved region (CRP), eight predicted transmembrane regions, a conserved zinc finger motif (CX_2_CX*n*CX_2_CX*n*CX_2_C) (Figure 2) and the conserved D, D, D, QxxRW signature sequence (D, D, D, QVLRW) involved in substrate binding and catalysis during cellulose synthesis (Figure 1). The predicted plant-specific conserved and hypervariable regions (HVRs) shared only approximately 16% to 35% identity. Thus, all these genes are predicted to be true *CesA* genes rather than cellulose synthase-like (CSL) genes as defined by Richmond and Somerville [10].

### 2.3. Phylogenetic Analysis of BplCesA Sequences

**Figure 3:**
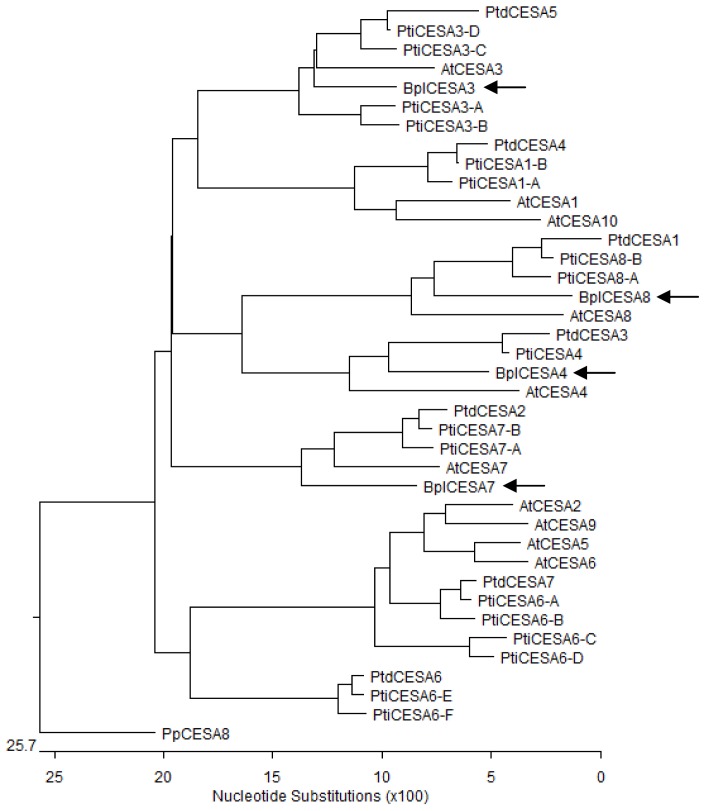
Phylogenetic relationships of deduced *Arabidopsis; Populus* and *Betula* CESA proteins. To identify the species of origin for each CESA, a species name or acronym is included before the name of the sequences: Pp: *Physcomitrella patens; At; Arabidopsis thaliana; Bpl; Betula platyphylla* (by arrow); Ptd; *Populus tremuloides; Pti; Populus trichocarpa*. The GenBank accession numbers are as follows: PpCESA8 (DQ902549); AtCESA1 (At4g32410); AtCESA2 (At4g39350); AtCESA3 (At5g05170); AtCESA4 (At5g44030); AtCESA5 (At5g09870). AtCESA6 (At5g64740); AtCESA7 (At5g17420); AtCESA8 (At4g18780); AtCESA9 (At2g21770); AtCESA10 (At2g25540); BplCESA8 (EU591529); BplCESA3 (EU591530); BplCESA7 (EU591531); BplCESA4 (EU591532); PtdCESA1 (AF072131); PtdCESA2 (AY095297); PtdCESA3 (AF527387); PtdCESA4 (AY162181); PtdCESA5 (AY055724); PtdCESA6 (AY196961); PtdCESA7 (AY162180); PtiCESA1-A (Pti835809); PtiCESA1-B (Pti763479); PtiCESA3-A (Pti560520); PtiCESA3-B (Pti576348); PtiCESA3-C (Pti821409); PtiCESA3-D (Pti706420); PtiCESA4 (Pti553321); PtiCESA6-A (Pti207792); PtiCESA6-B (Pti819877); PtiCESA6-C (Pti818594); PtiCESA6-D (Pti551308); PtiCESA6-E (Pti806784); PtiCESA6-F (Pti784751); PtiCESA7-A (Pti717644); PtiCESA7-B (Pti262611); PtiCESA8-A (Pti235238); PtiCESA8-B (Pti555650).

It is estimated that the *Populus* genome has 18 *CesA* genes [24,28,29], which can be grouped into eight sets of clearly defined paralogs, plus a single copy of *PtiCesA4* [23,28]. We used the 39 predicted full-lengh CESA amino acid sequences from five plant species, *Physcomitrella patens* (PpCESA8 as outgroup), *Arabidopsis thaliana* (10 AtCESAs), *P. trichocarpa* (17 PtiCESAs), *P. tremuloides* (7 PtdCESAs) and *B. platyphylla* (4 BplCESAs) to generate a phylogenetic tree (Figure 3). The multiple sequence alignment was shown in Figure S1. The two hypervariable regions (HVRI and HVRII) of the selected *CesA* gene products were highly divergent and excluded for the construction of the phylogenetic tree. The four *Betula* CESAs clustered with specific *Arabidopsis* and *Populus* CESA homologs (>80% identity) to form discrete clades (Figure 3). For example, BplCESA8 clustered with PtiCESA8-A/PtiCESA8-B (88.8%–89.2% amino acid sequence identity), AtCESA8 (86.6%) and PtdCESA1 (87.4%). BplCESA3 was considered orthologous to the PtiCESA3-C/PtiCESA3-D paralogs (92.8%–93.1% identity), while AtCESA3 (91.3%) and PtdCESA5 (89.6%). BplCESA7 shared 89.7% to 89.3% identity with PtiCESA7-A/PtiCESA7-B, 85.0% with AtCESA7 and 89.2% with PtdCESA2. BplCESA4 clustered with the single set comprising PtiCESA4 (89.9% identity), AtCESA4 (86.1%) and PtdCESA3 (88.0%). Genes in *s*uch clusters presumably share a common evolutionary history in which an ancestral CESA existed before the divergence of *Betula*, *Arabidopsis* and *Populus*. This parallel numbering model suggests that the clustered CESAs share similar functions in the three species. In addition, three predicted protein sequences of the four BplCESAs shared a maximum identity of 97.8% to 99.2% with three CESA isoforms from *B. luminifera*, whereas the other two CESA homologs, BplCESA3 and BluCESA5, shared only 63.6% to 71.9% identity with other CESAs of the two *Betula* species (Data not shown). Further investigations are required to confirm that the diverged *CesA* genes in *Betula* species perform distinct roles in cellulose biosynthesis.

### 2.4. Real-Time RT-PCR Analysis of the Expression Profiles of *BplCesAs*

**Figure 4:**
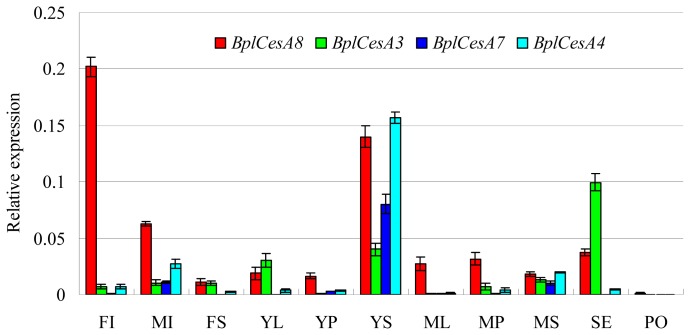
Quantitative real-time RT-PCR analysis of four *CesA* genes in tissues of *B. platyphylla*. The mRNA levels are expressed relative to the amount of *Bplactin* mRNA. FI: female inflorescence, MI: male inflorescence, FS: flower stalk, YL: young leaves, YP: young petiole, YS: young stem, ML: mid-development leaves, MP: mid-development petiole, MS: mid-development stem, SE: seed, PO: mature pollen. Data represent the mean ± standard error.

**Figure 5:**
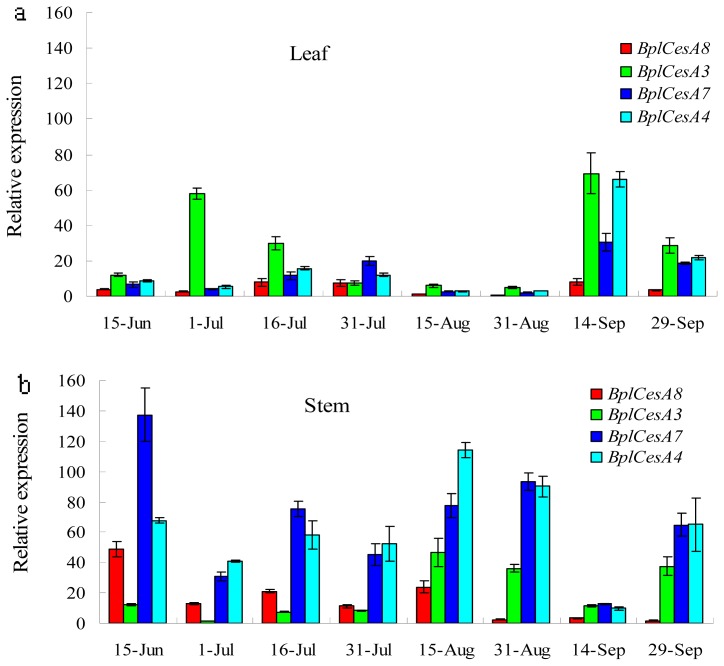
Quantitative real-time RT-PCR analysis of four *CesA* genes in leaves and stems at different developmental stages in *B. platyphylla*. The mRNA levels are normalized by *Bplactin* mRNA. Shown are individual mRNA abundances of the four *CesA* genes in leaves or stems. Data represent the mean ± standard error.

In order to research the transcript abundance and characteristics of the four novel *BplCesAs* in cell wall formation in distinct tissues, transcript expression profiles were generated in 11 different tissues of *Betula* by quantitative RT-PCR using gene-specific primers (Table 2). In our study, the abundance of all *BplCesA* mRNAs was detected and normalized to constitutively expressed *ACTIN* mRNA which has been improved to be qualified as a reference gene by Chao Dai *et al*. [30]. The results showed that *BplCesA8* was highly expressed in mature tissue, including some tissues with secondary thickening, such as male and female inflorescences (FI and MI), mature petioles as well as young and mid-development stem tissues rich in secondary cell wall (Figure 4). *BplCesA3* was predominantly expressed in tissues either typical of primary cell walls (young leaves in mid July) (Figure 4) or consistent with the expression pattern in early development (in July) of leaves as shown in Figure 5a and seeds (Figure 4). *BplCesA7* and *BplCesA4* were strictly co-expressed and expressed predominantly in developing and mature vascular tissues or tissues with secondary thickening, such as young and mature stems and male inflorescences. Another interesting result is that the four *BplCesAs* were expressed in mature pollen at an extremely low level compared with other tissues (Figure 4).

In addition, expression profiles for the four *BplCesAs* in developing leaves and stems were determined by quantitative RT-PCR using the same gene-specific primers (Table 2). Expression of *BplCesA* in stems was high during most developmental periods, with abundant expression of *BplCesA7* and *BplCesA4* (Figure 5). With the exception of *BplCesA3*, all *BplCesA*s were expressed at higher levels in stems than in leaves at most stages (Figure 5). *BplCesA8* mRNA was more abundant in stems than in leaves at earlier developmental stages, whereas *BplCesA3* mRNA was notably more abundant in leaves, which are richer in primary cell wall than stems at some time-points, especially earlier and later development stages (in July and September) (Figure 5). Both the *BplCesA7* and *BplCesA4* mRNAs were more abundant in stems (which are rich in secondary cell walls) than in leaves (which are typical of primary cell walls) during most of the examined periods (Figures 5).

## 3. Discussion

### 3.1. The *CesA* Family of *Betula* Comprises at Least Four Member Genes

No other CESA homologs have been identified in *B. platyphylla* in this study or elsewhere, indicating that the *CesA* gene family of *B. platyphylla* is comprised of at least four genes. It has been reported that the *CesA* gene family is comprised of 10 or more homologs in plants. For example, 93 cellulose synthesis-related genes have been identified in the *P. trichocarpa* genome, containing 17 *CesA* genes [23,24,28], 10 CESA homologs in *Arabidopsis* [12,23], and 11 *CesA* genes in rice [14]. There are 12 *CesA* gene members in *Pinus* [27]. The *CesA* gene family contains 11 *CesA* members in the moss (*Physcomitrella patens*) [31]. Here, only four *BplCesA*s were identified, which may be due to very low mRNA levels of other *BplCesA*s in certain tissues or developmental stages. Further studies are required to identify other *BplCesA*s in distinct tissues.

### 3.2. The Four BplCesAs Differentially Participate in the Development of Diverse Tissues in *Betula*

Each of the CESAs may have specific roles related to the formation of primary and secondary walls in plants. The distinct transcript expression profiles of the four *BplCesA*s indicated their different involvement in growth of *Betula*. Almost all of the four *BplCesAs* were highly expressed in the tissues we examined, indicating their involvement in the biosynthesis of cellulose which is the main component of plant cell walls. *BplCesA8* was more highly expressed in earlier growth stages of the stem (Figure 5) and there were more abundances of *BplCesA8* transcript in some tissues with secondary thickening (female and male inflorescences with abundant bracts and some mature tissues) or undergoing active secondary cell wall formation (in earlier growth as young stems) than other members (Figure 4). This indicated similarity to its homolog AtCESA8, which participates in secondary cell wall biosynthesis in *Arabidopsis* [11–13]. Similarly, PtiCESA8-A and PtiCESA8-B, which clustered with BplCESA8, also exhibited apparently higher xylem specificity [24]. This indicates BplCESA8 might be involved in secondary cell wall biosynthesis.

Notably we found high transcript expression levels of *BplCesA8* in male and female inflorescences (Figure 4). In *Arabidopsis*, CESA1 appears to be possibly required for either embryo or male gametophyte growth [32,33]. CESA3 is coexpressed with CESA1, and homozygous *cesa3* alleles are also male gametophyte lethal [13]. By comparison, in rice, OsCESA2,-9 were highly expressed in young panicle and the OsCSLF genes (OsCSLF2 & −7) were preferentially expressed in the hull of rice, while several CSL (cellulose synthase-like) genes (CSLG2,3 and CSLB2 in sepals, CSLG1, CSLD6 and CSLA1,2,10,11 in Carpels) are specifically expressed in flower organs in *Arabidopsis* [14]. The homologs of BplCESA8 were not reported to be related to the floral development either in rice or in *Arabidopsis*, suggesting *BplCesA8* may participate in floral growth in *Betula* unlike the other two plant species. Despite the involvement of *BplCesA8* in floral growth, there is no evidence for its involvement in male or female gametophyte growth because male and female inflorescences contain abundant bracts.

Also, we could assume *BplCesA8* to be a partially redundant candidate gene with *BplCesA3. BplCesA8* was detected with high transcript levels in specific tissues (male and female inflorescences, young stems) where the expression of *BplCesA3* is relatively low, while *BplCesA8* was lowly expressed in seed where *BplCesA3* showed much higher expression (Figure 4). In other words, *BplCesA3* may be partially redundant with *BplCesA8* in those specific tissues. Furthermore, the expression level of *BplCesA8* in female inflorescences very rich in bracts is much higher than that of *BplCesA3*, while *BplCesA3* was much more highly expressed than *BplCesA8* in developing seeds which were isolated from female inflorescences (Figure 4). The bracts in inflorescences are typical of secondary thickening cell walls. Thus, *BplCesA8* might be involved in the development of bracts and be related to secondary cell wall formation in *Betula* as with the result mentioned above.

Both *BplCesA7* and *BplCesA4* were expressed at apparently higher levels in the stem at most time-points, which are rich in secondary cell walls (Figures 4 and 5), indicating that the two genes may be involved in the biosynthesis of the secondary cell wall throughout the development of the stem. In addition, they were also more highly expressed in old leaves (in September) than young leaves. It is proposed that the changes in expression may reflect a role in the synthesis of homogalacturonan, which accumulates to a high level in old leaves [14]. But in *Arabidopsis*, it is cellulose synthase-like (CSL) genes (AtCSLD2 and AtCSLE1) which show sequence similarity to CESA apparently exhibiting strong increases in expression in old leaves versus young leaves instead of *CesA* genes [34]. This suggests the differences in both transcript expression and specific importance in different tissues of the members in the CES/CSL superfamily in the plant. More importantly, *BplCesA7* and *BplCesA4* were strictly co-expressed. OsCESA4, −7 (homologs of BplCESA4, −7, respectively) and -9 in rice are thought to be organized as a cellulose synthase complex involved in secondary cell wall synthesis [14], suggesting that the BplCESA7 and BplCESA4 may be related to the formation of a cellulose synthase complex for secondary cell wall biosynthesis. Similarly, the secondary cell wall development-related genes PtdCESA1, PtdCESA2 and PtdCESA3 in *P. tremuloides* [17–19] are all homologs of BplCESA8, BplCESA7, BplCESA4, respectively (Figure 3), suggesting that these three genes might be associated with secondary cell wall development in *Betula*. This is also consistent with the xylem-specific expression pattern of PtiCESA7-A or PtiCESA7-B (clustered with *BplCESA7*) and *PtiCESA*4 (clustered with *BplCESA4*) (Figure 3) in *P. trichocarpa* [24]. Redundant xylem-specific expression may suggest their involvement in the massive production of cellulose in xylem secondary cell walls for wood formation [25]. However, it has been reported that CesAs related to secondary cell walls are not functionally redundant, which suggests that AtCESA4 (a homolog of BplCESA4), AtCESA7 (of BplCESA7) and AtCESA8 (of BplCESA8) are the only CESAs involved in cellulose synthesis and form a complex in the secondary cell wall in *Arabidopsis* [35]. In spite of this, there is insufficient evidence in this research to support the participation of BplCESA8 (homolog of AtCESA8) complexed with BplCESA7 and BplCESA 4 (homologs of AtCESA7 and AtCESA4, respectively) for secondary cell wall formation.

BplCesA3 exhibited a unique and complex expression pattern (Figures 4 and 5). Although this gene was expressed at lower levels in most tissues compared with the other three BplCesAs, higher expression was detected at the earlier development in young leaves rich in primary cell wall (Figure 4 and Figure 5a). OsCESA1, −3 (homolog of BplCESA3), −5, −6, −8 in rice and AtCESA1, −2, −3 (homolog of BplCESA3), −5, −6 were detected at higher transcript expression levels in young seedlings mainly comprised of young leaves and stem. Also, OsCESA5/OsCESA6 is likely to be partially redundant with OsCESA3 for OsCESA complex organization in the young tissues, such as plumule and radicle [14]. AtCESA3 was reported to function predominantly in the primary cell wall. Furthermore, its homolog PtdCESA5 in *P. tremuloides* is involved in primary cell wall development together with PtdCESA4, PtdCESA6 and PtdCESA7 [21,22]. Usually, three or four CESAs are responsible for primary cell wall biosynthesis in higher plants. AtCESA1 [36], AtCESA 3 and AtCESA6 in Arabidopsis, OsCESA1, OsCESA3 and OsCESA8 in rice [14] and PtdCESA4, PtdCESA5, PtdCESA6 and PtdCESA7 in *P. tremuloides* [21,22] may form a cellulose synthase complex for primary cell wall biosynthesis in the three species. Therefore, it can be speculated that other primary cell wall related CESAs exist in *Betula* in addition to BplCESA3. In addition, the higher expression level in the late-development stem reflects a potential relation to the formation of secondary cell wall in *Betul*a. Finally, BplCesA3 was observed at highest expression level in old leaves (Figure 5a), which indicated that BplCesA3 may be related to the synthesis of homogalacturonan similar to BplCesA4, −7 in Betula.

Moreover, the transcript level of *BplCesA3* in developing seed was notably higher than in other tissues examined (Figure 4). During the development of seed, the secondary cell walls in the seed protect the embryo. Cellulose is proposed to play a major role in reinforcing the secondary cell wall in the seed coat epidermal cells [37] and to be a component of seed mucilage [38,39]. In *Arabidopsis thaliana*, AtCESA9 is required for normal secondary wall synthesis in epidermal seed coat cells [37]. AtCESA2, AtCESA5, and AtCESA9 subunits contribute to secondary wall synthesis in epidermal seed coat cells. AtCESA2 and AtCESA9 serve in radial wall reinforcement, as does CESA5, but CESA5 also functions in mucilage biosynthesis in the seed coat epidermis and is indispensable for mucilage attachment to the seed coat These data suggest unique roles for different CESA subunits in one cell type [40]. Though the homolog of BplCESA3, AtCESA3, has not been reported about its participation in the development of seed, AtCESA4, −7, −8, −9, −10 were detected highly expression levels in seed and silique [14]. The high transcript level of *BplCesA3* in developing seed reflects its possible involvement in development of seed in *Betula*. To sum up, the unique expression pattern of *BplCesA3* mentioned above indicated that it is possibly participating in diverse developments in *Betula* compared with the other three CESAs examined. Also, BplCESA3 might possess different transcript expression mechanisms in development of *Betula* from other plants.

We also found an unexpected result that almost each of the four *BplCesA3*, −*4*, −*7* and −*8* was observed at extremely low transcript level in mature pollen in *Betula* (Figure 4). It has been reported that AtCESA2, AtCESA6 and AtCESA9 are coexpressed during pollen development [11]. Triple *cesa2cesa6cesa9* mutant plants are pollen lethal, indicating that they function redundantly in pollen development. CESA9 has been proved to function redundantly with CESA6 during pollen development [13]. The extremely low transcript abundances of the four *BplCesAs* reflected their lower involvement in mature pollen development and that there would be other *CesAs* related to the maturity of pollen in *Betula*.

## 4. Materials and Methods

### 4.1. RT-PCR and Rapid Amplification of cDNA Ends (RACE) of Four *CesAs*

Young and mature leaves and stem tissues (after bark removal, the cambium and stem tissues were scraped with clean blades free of *RNase* contamination) were harvested from annual branches of mature birch trees growing in forests under natural conditions in the Northeast of China. Total RNA was extracted using the CTAB method and was treated with RNase-free DNase I (Promega, USA) to remove DNA contamination. The cDNA was synthesized from 2 μg total RNA using Oligo(dT) as the reverse primer in the reverse transcription-PCR (RT-PCR) system (Invitrogen, USA). The cDNA was used as a template for amplification in 50 μL PCR buffer (TaKaRa, China). Two upstream (U1 and U2) and two downstream (D1 and D2) degenerate primers (Table 2) were designed with Primer Premier 5.0 software based on conserved motif sequences of eight CESAs mRNAs from other plants [*Populus tremuloides CesA2* (AY095297), *Populus trichocarpa CesA6-E* (Pti806784), *Eucalyptus grandis CesA3* (DQ014507), *Arabidopsis thaliana CesA3* (At5g05170), *Bambusa oldhamii CesA5* (DQ020213), *Pinus taeda CesA3* (AY789652), *Pinus radiata CesA1* (AY639654), *Zea mays CesA12* (AY372246)]. PCR products were separated by agarose gel electrophoresis, and amplified products (approximately 2000-bp) were isolated and ligated into the pGEM-T easy vector (Promega, USA). 5′- and 3′-RACE were carried out with the SMART RACE cDNA amplification kit (Clontech, USA) employing gene-specific primers inferred from the PCR fragments (Table 2). The full-length sequences of *BplCesA*s were submitted to GenBank with the accession numbers EU591529, EU591530, EU591531 and EU591532.

### 4.2. Sequence Alignments and Phylogenetic Reconstruction

Open reading frames were identified by the ORF Finder tool [41,42]. Putative protein sequences were aligned using Clustal W2 with default parameters (BioEdit version 7.0.0.0; Tom Hall: Raleigh, NC, USA, 2005) [43] and MEGA 4.0 [44]. The alignments were edited with the BioEdit program. Transmembrane domains were identified and analyzed by TMpred [45]. The *Populus* genome assembly version 1.1 [29,46] was searched for CESA homologs. The phylogenetic tree was constructed based on the CLUSTAL W algorithm of MegAlign (version 5.01; DNAStar: Madison, WI, USA, 2001). For statistical analysis, 100 bootstrap replications were analyzed. The two hypervariable regions (HVRI and HVRII) of the selected *CesA* gene products were highly divergent and excluded for the construction of the multiple sequence alignment and phylogenetic tree. The largest part of the sequences containing the conserved cellulose synthase domain was selected to generate the tree, including CRP, eight predicted transmembrane regions, a conserved zinc finger motif and the conserved D, D, D, QxxRW signature sequence involved in substrate binding and catalysis during cellulose synthesis. Domain conservation was assessed by the conserved domain database (CDD Version 2.18; National Center for Biotechnology Information: Bethesda, MD, USA) in the NCBI website [47,48].

### 4.3. Real-Time RT-PCR Analysis

Real-time RT-PCR was carried out on RNA derived from young and old leaves and stems (eight developmental stages) of three 20-year-old mature *B. platyphylla* grown under natural conditions in the Northeast of China in 2007. Each sample was comprised of a pool of identical quantities of RNA from three birch plants. The cDNA corresponding to the HVRs (hypervariable regions) can be used as gene-specific primers or probes for molecular analyses [18,49,50]. Therefore, specific primer pairs (18–23 bp) (Table 2) were designed based on the two HVRs of the four *BplCesA* mRNAs and the specificity of the primers was tested by RT-PCR (Figure S2) and sequencing (Data not shown). The *B. platyphylla* Actin gene (GenBank accession number: EU588981) [51] was used as an internal control (reference gene) to normalize the amount of total RNA present in each reaction, which has been improved by Chao Dai *et al*. in 2011 [30]. The validity of the *Bplactin* gene as a control gene has been tested and proved by pre-experiment before the real-time RT-PCR (Figure S2). Experiments were carried out on a MJ OpticonTM2 machine (Bio-Rad, Hercules, CA) using the QuantiTect SYBR-green PCR Master Mix (TOYOBO, Osaka, Japan). All reactions were carried out in triplicate for technical and biological repetitions. PCR was performed in a 20 μL mixture consisting of 10 μL SYBR Premix Ex Taq, 2 μL cDNA, 1 μL of each gene specific (Table 1), and 6 μL dd H_2_O. The PCR amplification conditions were 95 °C for 5 min, followed by 40 cycles at 95 °C for 30 s, 60 °C for 30 s, 72 °C for 30 s, 79 °C for 1 s. Data from qRT-PCR experiments were analyzed by relative quantification according to [52]. The mRNA levels were normalized by the reference gene *Bplactin* mRNA. A melting curve was generated for each sample at the end of each run to assess the purity of the amplified products.

## 5. Conclusions

In this study, four members of the cellulose synthase (CESA) gene family were isolated and identified from a broadleaf tree species, *Betula platyphylla* Suk. The four putative BplCESA amino acid sequences contained the typical signature of the most processive glycosyltransferases and exhibited sequence identities ranging from 63.8% to 70.5%. Furthermore, the transcriptional expression level of the four *BplCesA*s was detected in diverse tissues and developmental periods were found to result in differential expression patterns. Results indicated that *BplCesA8* is related to secondary cell wall biosynthesis and floral development. *BplCesA3* is predominantly involved in primary cell wall biosynthesis and seed development, and also may be related to the synthesis of homogalacturonan, while BplCESA4 and BplCESA7 appear to participate in secondary cell wall biosynthesis and may be involved together in the formation of a cellulose synthase complex. These data indicate that these genes might be differentially involved in cellulose synthesis, cell wall development and diverse development in tissues in *Betula*. Further investigations are required to identify other *BplCesA* gene members and their biological functions in *Betula*.

## Supplementary Materials



## Figures and Tables

**Table 1:** Properties and main domains of four deduced BpCESA proteins.

Property	Symbol	Location in deduced protein sequence			

		BpCESA8	BpCESA3	BpCESA7	BpCESA4
Genbank accession	Genbank ACC	EU591529	EU591530	EU591531	EU591532
Full lengh (bp)		3255	3968	3399	3457
Untranslated region	5′-UTR	40	419	23	53
	3′-UTR	257	293	236	257
Open reading frame (bp)	ORF	2958	3255	3123	3147
Number of amino acid	aa	985	1084	1040	1048
Isoelectric point	p*I*	6.27	6.84	5.90	6.74
Molecular weight	MW (kDa)	110.4	121.2	117.7	119.5
transmembrane domain (TMDs)	TMD1	176–192	275–291	252–268	227–251
	TMD2	203–219	302–318	272–299	253–273
	TMD3	765–783	855–882	814–840	830–847
	TMD4	794–812	895–912	850–869	859–877
	TMD5	829–848	929–955	886–912	894–919
	TMD6	878–900	981–1003	937–958	945–965
	TMD7	911–932	1015–1033	971–989	976–997
	TMD8	943–960	1047–1063	1003–1019	1007–1026
Zn finger (Zn) CxxC	Zinc finger 46 bp	9–54	20–65	37–82	41–86
hypervariable region (HVR)	HVRI	55–174	66–273	83–250	87–224
	HVRII	545–669	644–770	620–726	594–735
plant conserved region (CRP)	CRP 125bp	301–425	400–524	377–501	351–474
UDP-glucose binding and catalysis sites (U)	U1 16bp	285–300	384–399	361–376	335–350
	U2 18bp	455–472	554–571	530–547	504–521
	U3 22bp	678–699	779–800	735–756	744–765
	U4 18bp	716–733	817–834	773–790	782–799
% Similarity to	BpCESA8	/	64.4	63.8	66.6
	BpCESA3	/	/	70.5	65.6
	BpCESA7	/	/	/	67.3

**Table 2:** Degenerate primers and specific primers for isolation and quantitative real-time PCR of *BplCesAs*.

Primers	Primer 5′→3′	Tm (°C)
*U1*	5′-TGGATTYTGGATCAGTTCCC-3′	
*U2*	5′-TGGATTYTDGATCAGTTCCC-3′	
*D1*	5′-TTVCCRAANAGMGGACCCCA-3′	
*D2*	5′-CCCATSAGACCYTTGAGGAA-3′	
*BplactinR*	TCA AGT TCC TGC TCA TAG TCA A	55.3
*BplactinF*	TTG CTA TCC AGG CTG TTC TC	55.3
*BplCesA8R*	TGC TCC ATA CGA TGA CGA CT	56.0
*BplCesA8F*	CCT TCC ATC TGC TGC TCT G	56.1
*BplCesA3R*	TGT CTG CTG CAT CAC CTG A	55.5
*BplCesA3F*	AAA GAG TCA TCC ACA AGC ACA T	56.4
*BplCesA7R*	GTA ATA GCC GGT GGT AGA TCC	55.9
*BplCesA7F*	TGC TCG AAG CAAT CGG TA	55.5
*BplCesA4R*	AGG CAG CAT GTC ACT ATC CA	55.3
*BplCesA4F*	TTC TTG CCT GAC TTT CCA CTT C	56.5

## Abbreviations

CESA: Cellulose synthase
cDNA: Complementary DNA
EST: Expressed Sequence Tag
NCBI: National Center for Biotechnology Information
ORF: Open Reading Frame
CDD: the Conserved Domain Database
TMDs: Transmembrane Domains
HVR: Hypervariable Regions
CRP: the Plant Conserved Region
QRT-PCR: Quantitative Reverse Transcription Polymerase Chain Reaction
RACE: Rapid Amplification of cDNA ends
UDP: Uridine 5′-diphosphate
Csl: CESA-like
CSR: the Class-Specific Region
pI: Isoelectric Point
TMHMM: Transmembrane helix prediction
DEPC: Diethylpyrocarbonate
CTAB: Cetyltrimethyl Ammonium Bromide
