# Supplementary material for: Four Novel Cellulose Synthase (CESA) Genes from Birch (Betula platyphylla Suk.) Involved in Primary and Secondary Cell Wall Biosynthesis
Source: Int J Mol Sci. 2012 Sep 25;13(10):12195–212. doi: 10.3390/ijms131012195 (PMC3497266; doi:10.3390/ijms131012195)

Supplemental Materials

Figure S1. Multiple sequence alignment and main domains for the deduced amino acid sequences of 39 CESA proteins from *Physcomitrella paten, Arabidopsis thaliana, Betula platyphylla*, *Populus tremuloides* and *Populus trichocarpa*. The two hypervariable regions (HVRI and HVRII) of the selected CesA gene products were highly divergent and excluded for the alignment. Shown are the conserved processive glycosyltransferase motif (D, D, D, QVLRW, black triangles). The GenBank accession numbers are as follows: PpCESA8(DQ902549), AtCESA1(At4g32410), AtCESA2(At4g39350), AtCESA3(At5g05170), AtCESA4(At5g44030), AtCESA5 (At5g09870). AtCESA6(At5g64740), AtCESA7(At5g17420), AtCESA8(At4g18780), AtCESA9(At2g21770), AtCESA10(At2g25540), BplCESA8 (EU591529), BplCESA3 (EU591530), BplCESA7 (EU591531), BplCESA4 (EU591532), PtdCESA1 (AF072131), PtdCESA2 (AY095297), PtdCESA3 (AF527387), PtdCESA4 (AY162181), PtdCESA5 (AY055724), PtdCESA6 (AY196961), PtdCESA7 (AY162180), PtiCESA1-A (Pti835809), PtiCESA1-B (Pti763479), PtiCESA3-A (Pti560520), PtiCESA3-B (Pti576348), PtiCESA3-C (Pti821409), PtiCESA3-D (Pti706420), PtiCESA4 (Pti553321), PtiCESA6-A (Pti207792), PtiCESA6-B (Pti819877), PtiCESA6-C (Pti818594), PtiCESA6-D (Pti551308), PtiCESA6-E (Pti806784), PtiCESA6-F (Pti784751), PtiCESA7-A (Pti717644), PtiCESA7-B (Pti262611), PtiCESA8-A (Pti235238), PtiCESA8-B (Pti555650).

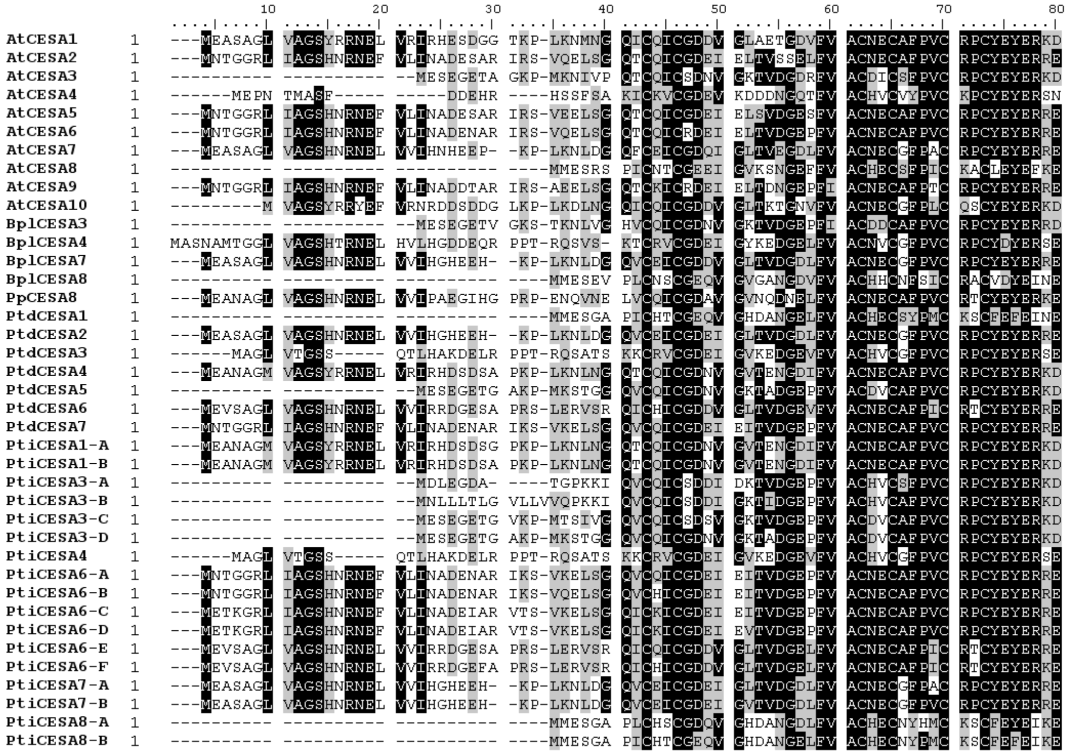

```
                                        90         100        110        120        130        140        150        160
                          ....|....| ....|....| ....|....| ....|....| ....|....| ....|....| ....|....| ....|....|
AtCESA1        77   GTQCCPQCTP YRVVIILRLI ILCFFLQYRT THPVKNAYPL WLTSVICEIW FAFSWLLDQF PKWYPINRET YLDRLAIRYD
AtCESA2        77   GNQACPQCNP YRMLILCRLA ILGLFFHYRI LHPVNDAYGL WLTSVICEIW FAVSWILDQF PKWYPIERET YLDRLSLRYE
AtCESA3        58   GNQSCPQCNP YRMVIMLRLV ILCLFLHYRI TNPVPNAFAL WLVSVICEIW FALSWILDQF PKWFPVNRET YLDRLALRYD
AtCESA4        61   GNKCCPQCSP YRIVIVLRLV ILVFFFRFRI LTPAKDAYPL WLISVICEIW FALSWILDQF PKWFPINRET YLDRLSMRFE
AtCESA5        77   GNQSCPQCNP YRMLIVLRLV ILGLFFHYRI LHPVNDAYAL WLISVICEIW FAVSWVLDQF PKWYPIERET YLDRLSLRYE
AtCESA6        77   GNQACPQCNP YRMLIVLRLV ILGLFFHYRI LHPVKDAYAL WLISVICEIW FAVSWVLDQF PKWYPIERET YLDRLSLRYE
AtCESA7        75   GTQNCPQCNP YRMVIVARLV ILAVFLRYRL LNPVHDALGL WLTSVICEIW FAVSWILDQF PKWFPIERET YLDRLSLRYE
AtCESA8        47   GRRICLRCTS YRIVIIMRLI ILALFFNYRI THPVDSAYGL WLTSVICEIW FAVSWVLDQF PKWSPINRET YIDRLSARFE
AtCESA9        77   GNQACPQCNP YRMLIFCRLA ILGLFFHYRI LHPVNDAFGL WLTSVICEIW FAVSWILDQF PKWYPIERET YLDRLSLRYE
AtCESA10       71   GSQCCPQCTP YRIVIVLRLI ILGVFLHYRT THPVKDAYAL WLTSVICEIW FAFSWLLDQF PKWYPINRET FLDRLALRYD
BplCESA3       58   GNQSCPQCNP YRMVIVLRLV VLSIFLHYRL TNPVPNACAL WLISVICEIW FAISWILDQF PKWLPVNRET YLDRLSLRYD
BplCESA4       79   GNQSCPQCSP YRIVIVLRLI ILAFFLRFRV LTPAYDAYPL WIISVICETW FAFSWILDQF PKWFPITRET YLDRLSMRFE
BplCESA7       75   GSQLCPQCNP YRMVIIARLV VLSLFLRYRL MNPVQDAFGL WLTSVICEIW FAISWILDQF PKWYPIDRET YLDRLSLRYE
BplCESA8       47   GRKACLRCTP YRCVIIMRLV ILSLFFHYRI THPVDSAFGL WLTSVICEIW FAVSWVLDQF PKWSPVERIT FTDELSARYE
PpCESA8        77   GNGVCPHCNP YRMVIVIRLV VLAFFLRYRI LHPVEGAFGL WITSVVCEIW FAVSWILDQF PKWLPIQRET YLDRLSLRYE
PtdCESA1       47   GRKVCLRCTP YRAVIIMRLV ILGLFFHFRI TNPVDSAFGL WLTSVICEIW FAFSWVLDQF PKWNPVNRET YIERLSARYE
PtdCESA2       75   GTQNCPQCNP YRMVIVARLI ILAVFLRYRI LHPVHDALGL WLTSIVCEIW FAISWILDQF PKWLPIDRET YLDRLSLRYE
PtdCESA3       69   GNQSCPQCNP YRIVIVLRLI ILCFFFRFWI LTPASDAYAL GLISVICEVW FGLSWILDQF PKWNPIERET YLDRLSMRFE
PtdCESA4       77   GTQSCPQCTP YRVGIILRLI ILGFFLQYRV THPVKDAYGL WLTSVICEIW FALSWLLDQF PKWMPINRET YLDRLALRYD
PtdCESA5       58   GNQSCPQCNP YRMVIVLRLG ILCIFLHYRI TNPVRNAYAL GLYLGIWGDW FAISRILDQF PKWLPGNRET YLDRLALRYD
PtdCESA6       77   GNQVCPQCNP YRMIIIVRLV VVGFFFHYRV THPVNDAFAL WLISVICEIW FAVSWILDQF PKWLPIDRET YLDRLSLRYE
PtdCESA7       77   GNQACPQCNP YRMIIILRLV IIGIFFHYRI LHPVNDAYGL WLTSVICEIW FAVSWILDQF PKWYPIERET YLDRLSLRYE
PtiCESA1-A     77   GTQSCPQCTP YRVVIILRLI ILGFFLQYRV THPVKDAYGL WLTSVICEIW FALSWLLDQF PKWMPINRET YLDRLALRYD
PtiCESA1-B     77   GTQSCPQCTP YRVVIILRLI ILGFFLQYRV THPVKDAYGL WLTSVICEIW FALSWLLDQF PKWMPINRET YLDRLALRYD
PtiCESA3-A     54   GNQSCPQCNP YRMVIVLRLV VLCIFLHYRL TNPVRDAYAL WLISVICEIW FAISWILDQF PKWLPVNRET YLDRLSLRYE
PtiCESA3-B     59   GNQSCPQCNP YRMVIVLRLV VLCIFLHYRL TNPVRNAYAL WLISVICEIW FAISWILDQF PKWLPVNRET YLDRLSLRYE
PtiCESA3-C     58   GNQSCPQCNP YRMVIVLRLI ILCIFLHYRI TNPVPNAYAL WLISVICEIW FAISWILDQF PKWLPVNRET YLDRLALRYE
PtiCESA3-D     58   GNQSCPQCNP YRMVIVLRLV ILCIFLHYRI TNPVRNAYAL WLISVICEIW FAISWILDQF PKWLPVNRET YLDRLALRYD
PtiCESA4       69   GNQSCPQCNP YRIVIVLRLI ILCFFFRFRI LTPAYDAYAL WLISVICEVW FGLSWILDQF PKWNPIERET YLDRLSMRFE
PtiCESA6-A     77   GNQACPQCNP YRMIIILRLV ILGIFFHYRI LHPVNDAYGL WLTSVICEIW FGVSWILDQF PKWYPIERET YLDRLSLRYE
PtiCESA6-B     77   GNQACPQCNP YRMIIILRLV VVGLFFHYRI LHPVNDAYGL WLTSVICEIW FAVSWILDQF PKWYPIERET YLDRLSLRYE
PtiCESA6-C     77   GNQACPQCSP YRLIIILRLV ILSLFFHYRI LHPVNDAYGL WLTSVICEIW FAISWILDQF PKWFPIERET YLDRLSLRYE
PtiCESA6-D     77   GNQACPQCSP YRLIIILRLV ILGLFFHYRI LHPVNDAYGL WLTSVICEIW FAVSWILDQF PKWYPIERET YLDRLSLRYE
PtiCESA6-E     77   GNQVCPQCNP YRMIIIVRLV VVGFFFHYRV THPVNDAFAL WLISVICEIW FAVSWILDQF PKWLPIDRET YLDRLSLRYE
PtiCESA6-F     77   GNQVCPQCNP YRMIIIIRLV VLGFFFHYRV THPVNDAFAL WLISVICEIW FAVSWILDQF PKWLPIDRET YLDRLSLRYE
PtiCESA7-A     75   GTQNCPQCNP YRMVIVARLI ILAFFLRYRI LHPVHDAIGL WLTSIVCEIW FAISWILDQF PKWLPIDRET YLDRLSLRYE
PtiCESA7-B     75   GTQNCPQCNP YRMVIVARLI ILAVFLRYRI LHPVHDAIGL WLTSIVCEIW FAISWILDQF PKWLPIDRET YLDRLSLRYE
PtiCESA8-A     47   GRKVCLRCTP YRAVIIMRLI ILGLFFHYRI TNPVDSAFGL WLTSVICEIW FAFSWVLDQF PKWNPVNREA FIDRLSARYE
PtiCESA8-B     47   GRKVCLRCTP YRAVIIMRLV ILGLFFHYRI TNPVDSAFGL WLTSVICEIW FAFSWVLDQF PKWNPVNRET YIERLSARYE

                                       170        180        190        200        210        220        230        240
                          ....|....| ....|....| ....|....| ....|....| ....|....| ....|....| ....|....| ....|....|
AtCESA1       157   RDGEPSQLVP VDVFVSTVDP LKEPPLVTAN TVLSILSVDY PVDKVACYVS DDGSAMLTFE SLSETAEFAK KWVPFCKKFN
AtCESA2       157   KEGKPSGLAP VDVFVSTVDP LKEPPLITAN TVLSILAVDY PVDKVACYVS DDGAAMLTFE ALSDTAEFAR KWVPFCKKFN
AtCESA3       138   REGEPSQLAA VDIFVSTVDP LKEPPLVTAN TVLSILAVDY PVDKVSCYVS DDGAAMLSFE SLAETSEFAR KWVPFCKKYS
AtCESA4       141   RDGEKNKLAP VDVFVSTVDP LKEPPIITAN TILSILAVDY PVNKVSCYVS DDGASMLLFD TLSETSEFAR RWVPFCKKYN
AtCESA5       157   KEGKPSELAG VDVFVSTVDP MKEPPLITAN TVLSILAVDY PVDRVACYVS DDGAAMLTFE ALSETAEFAR KWVPFCKKYT
AtCESA6       157   KEGKPSGLSP VDVFVSTVDP LKEPPLITAN TVLSILAVDY PVDKVACYVS DDGAAMLTFE ALSETAEFAR KWVPFCKKYC
AtCESA7       155   REGEPNMLAP VDVFVSTVDP LKEPPLVTSN TVLSILAMDY PVEKISCYVS DDGASMLTFE SLSETAEFAR KWVPFCKKFS
AtCESA8       127   REGEQSQLAA VDFFVSTVDP LKEPPLITAN TVLSILALDY PVDKVSCYVS DDGAAMLSFE SLVETADFAR KWVPFCKKYS
AtCESA9       157   KEGKPSELAP VDVFVSTVDP LKEPPLITAN TVLSILAVDY PVEKVACYVS DDGAAMLTFE ALSYTAEFAR KWVPFCKKFS
AtCESA10      151   RDGEPSQLAP VDVFVSTVDP MKEPPLVTAN TVLSILAVDY PVDKVACYVS DDGSAMLTFE ALSETAEFSK KWVPFCKKFN
BplCESA3      138   REGEVSQLAA VDIFVSTVDP LKEPPLVTAN TVLSILAVDY PVDKVSCYVS DDGAAMLTFE ALSETSEFAR KWVPFSKKYN
BplCESA4      159   REGEPNRLSP VDVFVSTVDP LKEPPIITAN TVLSILSVDY PVDKVSCYVS DDGASMLLFD TLSETAEFAR RWVPFCRKYS
BplCESA7      155   REGEPNQLAS VDLFVSTVDP MKEPPLVTAN TVLSILAMDY PVDKISCYIS DDGASMLTFE ALSETAEFAR KWVPFCKKFS
BplCESA8      127   REGEPSELAA VDFFVSTVDP LKEPPLITGN TVLSILAVDY PVDKVSCYVS DDGAAMLTFE SLVETADFAR KWVPFCKKFA
PpCESA8       157   KPGEPSQLVN VDVYVSTVDP LKEPPIVTAN TILSILAVDY PVDKVSCYLS DDGAAMLTFE ALSETSEFAR KWVPFCKKFT
PtdCESA1      127   REGEPSQLAG VDFFVSTVDP LKEPPLITAN TVLSILAVDY PVDKVSCYVS DDGAAMLSFE SLVETAEFAR KWVPFCKKFS
PtdCESA2      155   QEGGPNMLAP VDVFVSTVDP MKEPPLVTGN TLLSILAMDY PVEKISCYLS DDGASMCTFE AMSETAEFAR KWVPFCKKFN
PtdCESA3      149   REGEPNRLGP VDVFVSTVDP LKEPPIITAN TVLSILSVDY PVDKVSCYVS DDGASMLLFD SLAETAEFAR RWVPFCKKHN
PtdCESA4      157   RDGEPSQLAP IDIFVSTVDP MKEPPIVTAN TVLSILAVDY PVDKVSCYVS DDGSAMLTFE ALSETAEFAR KRRLSCKKHN
PtdCESA5      138   MEGEPSHLVV VDIFARSGVH LKEPPLVPAN AVLSILAGDS PIDKVSCYVS DDGAAMLTFE ALSETSEFSR KWVPFCKKYS
PtdCESA6      157   KEGQVSQLCP VDIYVSTVDP LKEPPLVTAN TVLSILAVDY PVDKVSCYVS DDGAAMLTFE ALSETSEFAK KWVPFCKKFS
PtdCESA7      157   KEGKPSELAS VDVFVSTVDP MKEPPLITAN TVLSILAVDY PVDKVACYVS DDGAAMLTFE ALSETSEFAR KWVPFCKKFN
PtiCESA1-A    157   REGEPSQLAP IDIFVSTVDP MKEPPLVTAN TVLSILAVDY PVDKVSCYVS DDGSAMLTFE ALSETAEFAR KWVPFCKKHS
PtiCESA1-B    157   RDGEPSQLAP IDIFVSTVDP LKEPPIVTAN TVLSILAVDY PVDKVSCYVS DDGSAMLTFE ALSETAEFAR KWVPFCKKHN
PtiCESA3-A    134   KEGEPSQLAA VDIFVSTVDP LKEPPLVTAN TVLSILAVDY PVDKVSCYVS DDGAAMLTFE AISETSEFAR KWVPFCKKYD
PtiCESA3-B    139   KEGEPSQLAA VDIFVSTVDP LKEPPLVTAN TVLSILAVDY PVDKVSCYVS DDGAAMLTFE TMSETSEFAR KWVPFCKRYD
PtiCESA3-C    138   HEGEPSQLAA VDIFVSTVDP LKEPPLVTAN TVLSILAVDY PIDKVSCYVS DDGAAMLTFE ALSETSEFAR KWVPFCKKYN
PtiCESA3-D    138   NEGEPSQLAA VDIFVSTVDP LKEPPLVTAN TVLSILAVDY PIDKVSCYVS DDGAAMLTFE ALSETSEFAR KWVPFCKKYS
PtiCESA4      149   REGEPNRLGP VDVFVSTVDP LKEPPIITAN TVLSILSVDY PVDKVSCYVS DDGASMLLFD SLAETAEFAR RWVPFCKKHN
PtiCESA6-A    157   KEGKPSELAS VDIFVSTVDP MKEPPLITAN TVLSILAVDY PVDKVACYVS DDGAAMLTFE ALSETSEFAR KWVPFCKKFN
PtiCESA6-B    157   KEGKPSELAS VDVFVSTVDP MKEPPLITAN TVLSILAVDY PVDKVACYVS DDGAAMLTFE ALSETSEFAR KWVPFCKKFN
PtiCESA6-C    157   KEGKPSELAS VDVFVSTVDP MKEPPLITAN TVLSILAVDY PVEKVACYVS DDGAAMLTFE AISETSEFAR KWVPFCKRFS
PtiCESA6-D    157   KEGKPSELAS VDVFVSTVDP MKEPPLITAN TVLSILAVDY PVDKVACYVS DDGAAMLTFE AISETSEFAR KWVPFCKRFS
PtiCESA6-E    157   KEGQASQLCP VDIYVSTVDP LKEPPLVTAN TVLSILAVDY PVDKVSCYVS DDGAAMLTFE ALSETSEFAK KWVPFCKKFS
PtiCESA6-F    157   KEGQPSQLSP VDIYVSTVDP LKEPPLVTAN TVLSILAVDY PVDKISCYVS DDGAAMLTFE ALSETSEFAK KWVPFCKKFS
PtiCESA7-A    155   REGEPNMLAP ADIFVSTVDP MKEPPLVTGN TILSILAMDY PVEKISCYLS DDGASMCTFE AMSETAEFAR KWVPFCKKYS
PtiCESA7-B    155   KEGEPNMLAP VDIFVSTVDP MKEPPLVTGN TLLSILAMDY PVEKISCYLS DDGASMCTFE AMSETAEFAR KWVPFCKKFN
PtiCESA8-A    127   REGEPSQLAA VDFFVSTVDP LKEPPLITAN TVLSILAVDY PVDKVSCYVS DDGAAMLTFE SLVETAEFAR KWVPFCKKFS
PtiCESA8-B    127   REGEPSQLAG VDFFVSTVDP LKEPPLITAN TVLSILAVDY PVDKVSCYVS DDGAAMLSFE SLVETAEFAR KWVPFCKKYT
                                                                             ▲
```

```
                             250        260        270        280        290        300        310        320
                       ....|....| ....|....| ....|....| ....|....| ....|....| ....|....| ....|....| ....|....|
AtCESA1       237  IEPRAPEFYF AQKIDYLKDK IQPSFVKERR AMKREYEEFK VRINALVAKA QKIPEEGWTM QDGTPWPGNN TRDHPGMIQ-
AtCESA2       237  IEPRAPEWYF SQKMDYLKNK VHPAFVRERR AMKRDYEEFK VKINALVATA QKVPEEGWTM QDGTPWPGNN VRDHPGMIQ-
AtCESA3       218  IEPRAPEWYF AAKIDYLKDK VQTSFVKDRR AMKREYEEFK IRINALVSKA LKCPEEGWVM QDGTPWPGNN TRDHPGMIQ-
AtCESA4       221  VEPRAPEFYF SEKIDYLKDK VQTTFVKDRR AMKREYEEFK VRINALVAKA QKKPEEGWVM QDGTPWPGNN TRDHPGMIQ-
AtCESA5       237  IEPRAPEWYF CHKMDYLKNK VHPAFVRERR AMKRDYEEFK VKINALVATA QKVPEEGWTM QDGTPWPGNN VRDHPGMIQ-
AtCESA6       237  IEPRAPEWYF CHKMDYLKNK VHPAFVRERR AMKRDYEEFK VKINALVATA QKVPEDGWTM QDGTPWPGNS VRDHPGMIQ-
AtCESA7       235  IEPRAPEMYF TLKVDYLQDK VHPTFVKERR AMKREYEEFK VRINAQVAKA SKVPLEGWIM QDGTPWPGNN TKDHPGMIQ-
AtCESA8       207  IEPRAPEFYF SLKIDYLRDK VQPSFVKERR AMKRDYEEFK IRMNALVAKA QKTPEEGWTM QDGTSWPGNN TRDHPGMIQ-
AtCESA9       237  IEPRAPEWYF SQKMDYLKHK VDPAFVMERR AMKRDYEEFK VKINALVSVS QKVPEDGWTM QDGTPWPGNN VRDHPGMIQ-
AtCESA10      231  IEPRAPEFYF SQKIDYLKDK IQPSFVKERR AMKREYEEFK VRINILVAKA QKIPEDGWTM EDGTSWPGNN PRDHPGMIQ-
BplCESA3      218  IEPRAPEWYF AQKVDYLKDK VQTSFVKERR AMKREYEEFK VRVNALVAKA QKIPEEGWIM QDGTPWPGNN TRDHPGMIQ-
BplCESA4      239  IEPRAPEYYF SEKMDYLKDK VLPSFVKERR AMKREYEEFK VRINALVAKA QKKPEEGWVM QDGTPWPGNN TRDHPGMIQ-
BplCESA7      235  IEPRAPEMYF AEKIDYLKDK VQPTFVKERR AMKREYEEFK VRVNALVAKA TKVPPEGWIM QDGTPWPGNN TKDHPGMIQ-
BplCESA8      207  IEPRAPEFYF AQKIDYLKDK VQPSFVKERR AMKRDYEEFK VRVNALVAKA QKTPEEGWTM EDGTAWPGNN SRDHPGMIQ-
PpCESA8       237  IEPRAPEMYF AQKIDYLRDK VQPTFVKERR AMKREYEEFK VRVNALVAKA LKVPEDGWTM QDGTPWPGNN KSDHPGMIQ-
PtdCESA1      207  IEPRAPEFYF SQKIDYLKDK VQPSFVKERR AMKRDYEEYK VRVNALVAKA QKTPEEGWTM QDGTPWPGNN TRDHPGHDSG
PtdCESA2      235  IEPRAPEFYF TLKVDYLKDK VQPTFVKERR AMKREYEEFK VRINAIVAKA QKVPTEGWIM QDGTPWPGNN TRDHPGMIQ-
PtdCESA3      229  IEPRAPEFYF TQKIDYLKDK VHPNFVKERR AMKREYEEFK VRINALVSKA QKKPEEGWVM QDGTPWPGNI TRDHPGMIQ-
PtdCESA4      237  IEPRAPEFYF AQKMDYLEDK IQPSFVKERR AMKREYEEFK VRINALVAKA QKMPEEGWTM QDGTPWPGNN PRDHPGMIQ-
PtdCESA5      218  IEPRAPEWYF AQKIDYLKDK VQPSFVKDRR AMKREYEEFK IRINGLVAKA QKVPEEGWIM QDGTPWPGNN TRDHPGMIQ-
PtdCESA6      237  IEPRAPEFYF SQKIDYLKDK VQASFVKERR AMKREYEEFK IRINALVAKA HKVPEDGWTM QDGTPWPGNN VRDHPGMIQ-
PtdCESA7      237  IEPRAPEWYF SQKIDYLKNK VHPAFVRERR AMKREYEEFK VRINGLVSTA QKVPEDGWTM QDGTPWPGNN VRDHPGMIQ-
PtiCESA1-A    237  IEPRAPEFYF AQKIDYLKDK IQPSFVKERR AMKREYEEFK VRINALVAKA QKMPEEGWTM QDGTPWPGNN PRDHPGMIQ-
PtiCESA1-B    237  IEPRAPEFYF AQKIDYLKDK IQPSFVKERR AMKREYEEFK VRINALVAKA QKMPEEGWTM QDGTPWPGNN PRDHPGMIQ-
PtiCESA3-A    214  IEPRAPEWYF AQKIDYLKDK VHPAFVKERR AMKREYEEFK VRVNGFVSKA QKVPDEGWVM QDGTPWPGNN TRDHPGMIQ-
PtiCESA3-B    219  IEPRAPEWYF SQKIDYLKDK VHPSFVKERR AMKREYEEFK VRVNGLVAKA QKVPDEGWVM QDGTPWPGNN IRDHPGMIQ-
PtiCESA3-C    218  IEPRAPEFYF SQKIDYLKDK VQPSFVKDRR AMKREYEEFK IRVNGLVSKA QKVPEEGWIM QDGTPWPGNN TRDHPGMIQ-
PtiCESA3-D    218  IEPRAPEWYF AQKIDYLKDK VQPSFVKDRR AMKREYEEFK IRINGLVAKA QKVPEEGWIM QDGTPWPGNN TRDHPGMIQ-
PtiCESA4      229  IEPRAPEFYF TQKIDYLKDK VHPNFVKERR AMKREYEEFK VRINALVSKA QKKPEEGWVM QDGTPWPGNI TRDHPGMIQ-
PtiCESA6-A    237  IEPRAPEWYF SQKIDYLKNK VHPAFVRERR AMKREYEEFK VRINGLVSTA QKVPEDGWTM QDGTPWPGNN VRDHPGMIQ-
PtiCESA6-B    237  IEPRAPEWYF SQKMDYLKNK VHPAFVRERR AMKREYEEFK VKINGLVATA QKVPEDGWTM QDGTPWPGNN VRDHPGMIQ-
PtiCESA6-C    237  IEPRAPEWYF AKKVDYLKDK VDPAFIRERR AMKREYEEFK VRINGLVAMA QKVPEDGWTM QDGSPWPGNN VRDHPGMIQ-
PtiCESA6-D    237  IEPRAPEWYF AQKVDYLKDR VDPAFIRERR AMKREYEEFK VRINGLVATA QKVPEDGWTM QDGTPWPGNN VRDHPGMIQ-
PtiCESA6-E    237  IEPRAPEFYF AQKIDYLKDK VQASFVKERR AMKREYEEFK VRINALVSKA HKVPEDGWTM QDGTPWPGNN VRDHPGMIQ-
PtiCESA6-F    237  IEPRAPEFYF AQKIDYLKDK VDASFVKERR AMKREYEEFK VRVNALVAKA HKVPEDGWTM QDGTPWPGNN VRDHPGMIQ-
PtiCESA7-A    235  IEPRAPEFYF ALKIDYLKDK VQPTFVKERR AMKREYEEFK VRINAIVAKA QKVPPEGWIM QDGTPWPGNN TRDHPGMIQ-
PtiCESA7-B    235  IEPRAPEFYF TLKVDYLKDK VQPTFVKERR AMKREYEEFK VRINAIVAKA QKVPTEGWIM QDGTPWPGNN TRDHPGMIQ-
PtiCESA8-A    207  IEPRAPEFYF SQKIDYLKDK VQPSFVKERR AMKRDYEEYK VRVNALVAKA QKTPDEGWTM QDGTPWPGNN TRDHPGMIQ-
PtiCESA8-B    207  IEPRAPEFYF SLKIDYLKDK VQPSFVKERR AMKRDYEEYK VRVNALVAKA QKTPEEGWTM QDGTPWPGNN TRDHPGMIQ-

                             330        340        350        360        370        380        390        400
                       ....|....| ....|....| ....|....| ....|....| ....|....| ....|....| ....|....| ....|....|
AtCESA1       316  VFLGHSGGLD TDGNELPRLI YVSREKRPGF QHHKKAGAMN ALIRVSAVLT NGAYLLNVDC DHYFNNSKAI KEAMCFMMDP
AtCESA2       316  VFLGHSGVRD TDGNELPRLV YVSREKRPGF DHHKKAGAMN SLIRVSAVLS NAPYLLNVDC DHYINNSKAI RESMCFMMDP
AtCESA3       297  VFLGQNGGLD AEGNELPRLV YVSREKRPGF QHHKKAGAMN ALVRVSAVLT NGPFILNLDC DHYINNSKAL REAMCFLMDP
AtCESA4       300  VYLGKEGAFD IDGNELPRLV YVSREKRPGY AHHKKAGAMN AMVRVSAVLT NAPFMLNLDC DHYINNSKAI RESMCFLMDP
AtCESA5       316  VFLGNNGVRD VENNELPRLV YVSREKRPGF DHHKKAGAMN SLIRVSGVLS NAPYLLNVDC DHYINNSKAL REAMCFMMDP
AtCESA6       316  VFLGSDGVRD VENNELPRLV YVSREKRPGF DHHKKAGAMN SLIRVSGVLS NAPYLLNVDC DHYINNSKAL REAMCFMMDP
AtCESA7       314  VFLGHSGGFD VEGHELPRLV YVSREKRPGF QHHKKAGAMN ALVRVAGVLT NAPFMLNLDC DHYVNNSKAV REAMCFLMDP
AtCESA8       286  VFLGYSGARD IEGNELPRLV YVSREKRPGY QHHKKAGAEN ALVRVSAVLT NAPFILNLDC DHYVNNSKAV REAMCFLMDP
AtCESA9       316  VFLGHSGVCD MDGNELPRLV YVSREKRPGF DHHKKAGAMN SLIRVSAVLS NAPYLLNVDC DHYINNSKAI REAMCFMMDP
AtCESA10      310  VFLGHSGGLD TDGNELPRLI YVSREKRPGF QHHKKAGAMN ALIRVSAVLT NGAYLLNVDC DHYFNNSKAI KEAMCFMMDP
BplCESA3      297  VFLGQSGGLD AEGNELPRLV YVSREKRPGF QHHKKAGAMN ALVRVSAVLT NGPFLLNLDC DHYVNNSKAL REAMCFLMDP
BplCESA4      318  VIL-SEGALD VEGKELPRLV YVSREKRPGY QHHKKAGAMN ALVRVSAVLT NAPFTLNLDC DHYINNSKAV REAMCFLMDP
BplCESA7      314  VFLGHSGGVD AEGNELPRLV YVSREKRP-V STSQEAGAMN ALVRVSAVLT NAPFILNLDC DHYINNSKAA REAMCFLMDP
BplCESA8      286  VFLGSTGAHD IEGNELPRLV YVSREKRPGY QHHKKAGAEN ALVRVSAVLT NAPYILNLDC DHYVNNSKAV REAMCFLMDP
PpCESA8       316  VFLGHSGGLD TDGNELPRLV YVSREKRPGF NHHKKAGAMN ALVRVSAVLT NAPYMLNLDC DHYINNSKAI REAMCFMMDP
PtdCESA1      287  LPWEILGARD IEGNELPRLV YVSREKRPGY QHHKKAGAEN ALVRVSAVLT NAPYILNVDC DHYVNNSKAV REAMCILMDP
PtdCESA2      314  VFLGHSGGHD VEGNELPRLV YVSREKRPGF SHHKKAGAMN ALIRVLAILT NAPFMLNLDC DHYVNNSKAV REAMCFLMDP
PtdCESA3      308  VYLGSEGALD VEGKELPRLV YVSREKRPGY NHHKKAGAMN ALIRVSAVLT NAPFMLNLDC DHYINNSKAV REAMCFLMDP
PtdCESA4      316  VFLGHSGGLD TDGNELPRLV YVSREKRPGF QHHKKAGAMN ALIRVSAVLT NGAYLLNVDC DHYFNNSKAL KEAMCFMMDP
PtdCESA5      297  VFLGQSGGLD SDGNELPRLV YVSREKRPGF QHHKKAGAMN SLVRVSAVLT NGPFLLNLDC DHYINISKAL REAMCFMKDP
PtdCESA6      316  VFLGQSGGHD TDGNELPRLV YVSREKRPGF NHHKKAGAMN ALVRVSAVLT NAPYLLNLDC DHYINNSKAL REAMCFMMDP
PtdCESA7      316  VFLGQSGVRD VEGCELPRLV YVSREKRPGF EHHKKAGAMN SLMRVSAVLS NAPYLLNVDC DHYINNSRAL REAMCFMMDP
PtiCESA1-A    316  VFLGHSGGLD TDGNELPRLV YVSREKRPGF QHHKKAGAMN ALIRVSAVLT NGAYLLNVDC DHYFNNSKAL KEAMCFMMDP
PtiCESA1-B    316  VFLGHSGGLD TDGNELPRLV YVSREKRPGF QHHKKAGAMN ALIRVSAVLT NGAYLLNVDC DHYFNNSKAL KEAMCFMMDP
PtiCESA3-A    293  VFLGHSGGLD TEGNELPRLV YVSREKRPGF QHHKKAGAMN ALVRVSAVLT NGPFLLNLDC DHYINNSRAL REAMCFLMDP
PtiCESA3-B    298  VFLGHSGGLD TEGNELPRLV YVSREKRPGF QHHKKAGAMN ALVRVSAVLT NGPFLLNLDC DHYINNSKAL REAMCFLMDP
PtiCESA3-C    297  VFLGQSGGLD TDGNELPRLV YVSREKRPGF QHHKKAGAMN SLVRVSAVLT NGPFLLNLDC DHYINNSKAL REAMCFMMDP
PtiCESA3-D    297  VFLGQSGGLD SDGNELPRLV YVSREKRPGF QHHKKAGAMN SLVRVSAVLT NGPFLLNLDC DHYINNSKAL REAMCFMMDP
PtiCESA4      308  VYLGSEGALD VEGKELPRLV YVSREKRPGY NHHKKAGAMN ALIRVSAVLT NAPFMLNLDC DHYINNSKAV REAMCFLMDP
PtiCESA6-A    316  VFLGQSGVRD VEGCELPRLV YVSREKRPGF EHHKKAGAMN ALVRVSAVLS NAPYLLNVDC DHYINNSRAL REAMCFMMDP
PtiCESA6-B    316  VFLGQSGVRD VEGNELPRLV YVSREKRPGF EHHKKAGAMN ALMRVTAVLS NAPYLLNVDC DHYINNSRAL REAMCFLMDP
PtiCESA6-C    316  VFLGHNGVHD VEGNELPRLV YVSREKRPGF DHHKKAGAMN ALVRVSAIIS NAPYMLNVDC DHYINNSKAL REAMCFMMDP
PtiCESA6-D    316  VFLGHNGVHD VEGNELPRLV YVSREKRPGF DHHKKAGAMN SLVRVSAIIT NAPYMLNVDC DHYINNSKAL REAMCFMMDP
PtiCESA6-E    316  VFLGQSGGHD TDGNELPRLV YVSREKRPGF NHHKKAGAMN ALVRVSAVLT NAPYLLNLDC DHYINNSKAL REAMCFMVDP
PtiCESA6-F    316  VFLGQSGGHD TDGNELPRLV YVSREKRPGF NHHKKAGAMN ALVRVSAVLS NARYLLNLDC DHYINNSKAL RESMCFMMDP
PtiCESA7-A    314  VFLGHSGGHD TEGNELPRLV YVSREKRPGF SHHKKAGAMN ALIRVSAVLT NAPFMLNLDC DHYINNSKAV REAMCFLMDP
PtiCESA7-B    314  VFLGHSGGHD VEGNELPRLV YVSREKRPGF SHHKKAGAMN ALIRVSAVLT NAPFMLNLDC DHYVNNSKAV REAMCFLMDP
PtiCESA8-A    286  VFLGNTGARD IEGNELPRLV YVSREKRPGY QHHKKAGAEN ALVRVSAVLT NAPYILNLDC DHYVNNSKAV REAMCILMDP
PtiCESA8-B    286  VFLGNTGARD IEGNELPRLV YVSREKRPGY QHHKKAGAEN ALVRVSAVLT NAPYILNLDC DHYVNNSKAV REAMCILMDP
```

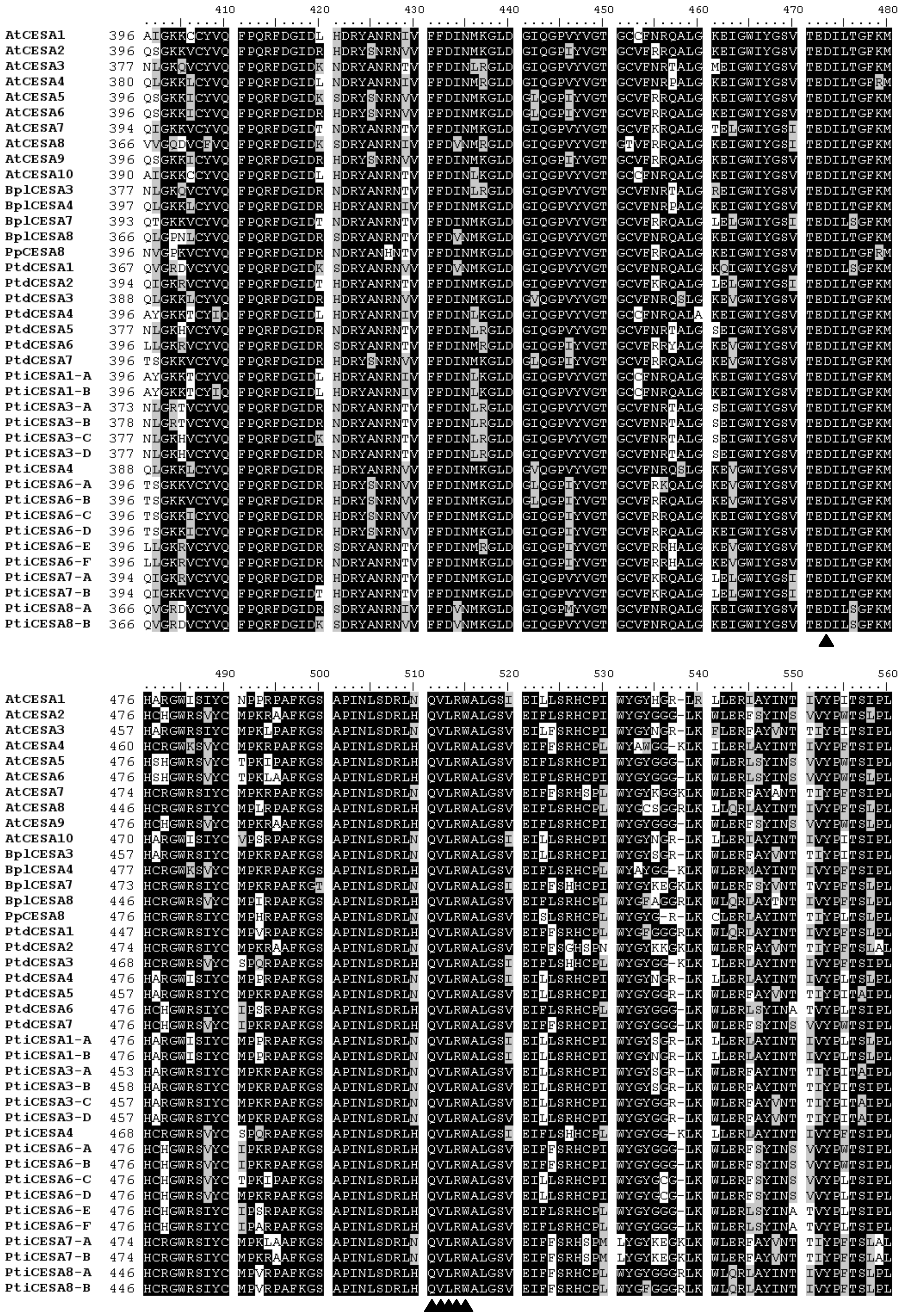
410 420 430 440 450 460 470 480
AtCESA1 396 AIGKKCCYVQ FPQRFDGIDL HDRYANRNIV FFDINMKGLD GIQGPVYVGT GCCFNRQALG KEIGWIYGSV TEDILTGFKM
AtCESA2 396 QSGKKVCYVQ FPQRFDGIDR HDRYSNRNVV FFDINMKGLD GIQGPIYVGT GCVFRRQALG KEIGWIYGSV TEDILTGFKM
AtCESA3 377 NLGKQVCYVQ FPQRFDGIDK NDRYANRNTV FFDINLRGLD GIQGPVYVGT GCVFNRTALG MEIGWIYGSV TEDILTGFKM
AtCESA4 380 QLGKKLCYVQ FPQRFDGIDL NDRYANRNIV FFDINMRGLD GIQGPVYVGT GCVFNRPALG KEIGWIYGSV TEDILTGFRM
AtCESA5 396 QSGKKICYVQ FPQRFDGIDK SDRYSNRNVV FFDINMKGLD GLQGPIYVGT GCVFRRQALG KEIGWIYGSV TEDILTGFKM
AtCESA6 396 QSGKKICYVQ FPQRFDGIDR HDRYSNRNVV FFDINMKGLD GLQGPIYVGT GCVFRRQALG KEIGWIYGSV TEDILTGFKM
AtCESA7 394 QIGKKVCYVQ FPQRFDGIDT NDRYANRNTV FFDINMKGLD GIQGPVYVGT GCVFKRQALG TELGWIYGSI TEDILTGFKM
AtCESA8 366 VVGQDVCFVQ FPQRFDGIDK SDRYANRNIV FFDVNMRGLD GIQGPVYVGT GTVFRRQALG KEIGWIYGSI TEDILTGFKM
AtCESA9 396 QSGKKICYVQ FPQRFDGIDR HDRYSNRNVV FFDINMKGLD GIQGPIYVGT GCVFRRQALG KEIGWIYGSV TEDILTGFKM
AtCESA10 390 AIGKKCCYVQ FPQRFDGIDL HDRYANRNTV FFDINLKGLD GIQGPVYVGT GCCFNRQALG KEIGWIYGSV TEDILTGFKM
BplCESA3 377 NLGKQVCYVQ FPQRFDGIDR NDRYANRNTV FFDINLRGLD GIQGPVYVGT GCVFNRTALG REIGWIYGSV TEDILTGFKM
BplCESA4 397 QLGKKLCYVQ FPQRFDGIDR HDRYANRNIV FFDINMKGLD GIQGPVYVGT GCVFNRPALG KEIGWIYGSV TEDILTGFKM
BplCESA7 393 QTGKKVCYVQ FPQRFDGIDT NDRYANRNTV FFDINMKGLD GIQGPVYVGT GCVFRRQALG LELGWIYGSI TEDILSGFKM
BplCESA8 366 QLGPNLCYVQ FPQRFDGIDR SDRYANRNTV FFDVNMKGLD GIQGPVYVGT GCVFNRQALG KEIGWIYGSV TEDILTGFKM
PpCESA8 396 NVGPKVCYVQ FPQRFDGIDR NDRYANHNTV FFDINMKGLD GIQGPVYVGT GCVFRRQALG KEIGWIYGSV TEDILTGFRM
PtdCESA1 367 QVGRDVCYVQ FPQRFDGIDK SDRYANRNVV FFDVNMKGLD GIQGPVYVGT GCVFNRQALG KQIGWIYGSV TEDILSGFKM
PtdCESA2 394 QIGKRVCYVQ FPQRFDGIDT HDRYANRNTV FFDINMKGLD GIQGPVYVGT GCVFKRQALG LELGWIYGSI TEDILTGFKM
PtdCESA3 388 QLGKKLCYVQ FPQRFDGIDR HDRYANRNVV FFDINMKGLD GVQGPVYVGT GCVFNRQSLG KEVGWIYGSV TEDILTGFKM
PtdCESA4 396 AYGKKTCYIQ FPQRFDGIDL HDRYANRNIV FFDINLKGLD GIQGPVYVGT GCCFNRQALA KEIGWIYGSV TEDILTGFKM
PtdCESA5 377 NLGKHVCYVQ FPQRFDGIDR NDRYANRNTV FFDINLRGLD GIQGPVYVGT GCVFNRTALG SEIGWIYGSV TEDILTGFKM
PtdCESA6 396 LLGKRVCYVQ FPQRFDGIDR SDRYANRNTV FFDINMRGLD GIQGPIYVGT GCVFRRYALG KEVGWIYGSV TEDILTGFKM
PtdCESA7 396 TSGKKVCYVQ FPQRFDGIDR HDRYSNRNVV FFDINMKGLD GLQGPIYVGT GCVFRRQALG KEVGWIYGSV TEDILTGFKM
PtiCESA1-A 396 AYGKKTCYVQ FPQRFDGIDL HDRYANRNIV FFDINLKGLD GIQGPVYVGT GCCFNRQALG KEIGWIYGSV TEDILTGFKM
PtiCESA1-B 396 AYGKKTCYIQ FPQRFDGIDL HDRYANRNIV FFDINLKGLD GIQGPVYVGT GCCFNRQALG KEIGWIYGSV TEDILTGFKM
PtiCESA3-A 373 NLGRTVCYVQ FPQRFDGIDR NDRYANRNTV FFDINLRGLD GIQGPVYVGT GCVFNRTALG SEIGWIYGSV TEDILTGFKM
PtiCESA3-B 378 NLGRTVCYVQ FPQRFDGIDR NDRYANRNTV FFDINLRGLD GIQGPVYVGT GCVFNRTALG SEIGWIYGSV TEDILTGFKM
PtiCESA3-C 377 NLGKHVCYVQ FPQRFDGIDK NDRYANRNTV FFDINLRGLD GIQGPVYVGT GCVFNRTALG SEIGWIYGSV TEDILTGFKM
PtiCESA3-D 377 NLGKHVCYVQ FPQRFDGIDR NDRYANRNTV FFDINLRGLD GIQGPVYVGT GCVFNRTALG SEIGWIYGSV TEDILTGFKM
PtiCESA4 388 QLGKKLCYVQ FPQRFDGIDR HDRYANRNVV FFDINMKGLD GVQGPVYVGT GCVFNRQSLG KEVGWIYGSV TEDILTGFKM
PtiCESA6-A 396 TSGKKVCYVQ FPQRFDGIDR HDRYSNRNVV FFDINMKGLD GLQGPIYVGT GCVFRKQALG KEVGWIYGSV TEDILTGFKM
PtiCESA6-B 396 TSGKKVCYVQ FPQRFDGIDR HDRYSNRNVV FFDINMKGLD GLQGPIYVGT GCVFRRQALG KEVGWIYGSV TEDILTGFKM
PtiCESA6-C 396 TSGKKICYVQ FPQRFDGIDR HDRYSNRNVV FFDINMKGLD GIQGPIYVGT GCVFRRQALG KEIGWIYGSV TEDILTGFKM
PtiCESA6-D 396 TSGKKICYVQ FPQRFDGIDR HDRYSNRNVV FFDINMKGLD GIQGPIYVGT GCVFRRQALG KEIGWIYGSV TEDILTGFKM
PtiCESA6-E 396 LLGKRVCYVQ FPQRFDGIDR SDRYANRNTV FFDINMRGLD GIQGPIYVGT GCVFRRHALG KEVGWIYGSV TEDILTGFKM
PtiCESA6-F 396 LLGKRVCYVQ FPQRFDGIDR NDRYANRNTV FFDINMKGLD GIQGPIYVGT GCVFRRHALG KEVGWIYGSV TEDILTGFKM
PtiCESA7-A 394 QIGKRVCYVQ FPQRFDGIDR HDRYANRNTV FFDINMKGLD GIQGPVYVGT GCVFKRQALG LELGWIYGSI TEDILTGFKM
PtiCESA7-B 394 QIGKKVCYVQ FPQRFDGIDT HDRYANRNTV FFDINMKGLD GIQGPVYVGT GCVFKRQALG LELGWIYGSI TEDILTGFKM
PtiCESA8-A 366 QVGRDVCYVQ FPQRFDGIDR SDRYANRNIV FFDVNMKGLD GIQGPMYVGT GCVFNRQALG KEIGWIYGSV TEDILSGFKM
PtiCESA8-B 366 QVGRDVCYVQ FPQRFDGIDK SDRYANRNVV FFDVNMKGLD GIQGPVYVGT GCVFNRQALG KEIGWIYGSV TEDILSGFKM
490 500 510 520 530 540 550 560
AtCESA1 476 HARGWISIYC NPPRPAFKGS APINLSDRLN QVLRWALGSI EILLSRHCPI WYGYHGR-LR LLERIAYINT IVYPITSIPL
AtCESA2 476 HCHGWRSVYC MPKRAAFKGS APINLSDRLH QVLRWALGSV EIFLSRHCPI WYGYGGG-LK WLERFSYINS VVYPWTSLPL
AtCESA3 457 HARGWRSIYC MPKLPAFKGS APINLSDRLN QVLRWALGSV EILFSRHCPI WYGYNGR-LK FLERFAYVNT TIYPITSIPL
AtCESA4 460 HCRGWKSVYC MPKRPAFKGS APINLSDRLH QVLRWALGSV EIFFSRHCPL WYAWGG-KLK ILERLAYINT IVYPFTSIPL
AtCESA5 476 HSHGWRSVYC TPKIPAFKGS APINLSDRLH QVLRWALGSV EIFLSRHCPI WYGYGGG-LK WLERLSYINS VVYPWTSIPL
AtCESA6 476 HSHGWRSVYC TPKLAAFKGS APINLSDRLH QVLRWALGSV EIFLSRHCPI WYGYGGG-LK WLERLSYINS VVYPWTSLPL
AtCESA7 474 HCRGWRSIYC MPKRPAFKGS APINLSDRLN QVLRWALGSV EIFFSRHSPL WYGYKGGKLK WLERFAYANT TIYPFTSIPL
AtCESA8 446 HCRGWRSIYC MPLRPAFKGS APINLSDRLH QVLRWALGSV EIFLSRHCPL WYGCSGGRLK LLQRLAYINT IVYPFTSLPL
AtCESA9 476 HCHGWRSVYC MPKRAAFKGS APINLSDRLH QVLRWALGSV EIFLSRHCPI WYGYGGG-LK WLERFSYINS VVYPWTSLPL
AtCESA10 470 HARGWISIYC VPSRPAFKGS APINLSDRLN QVLRWALGSI EILLSRHCPI WYGYNGR-LK LLERIAYINT IVYPITSIPL
BplCESA3 457 HARGWRSIYC MPKRPAFKGS APINLSDRLN QVLRWALGSV EILLSRHCPI WYGYSGR-LK WLERFAYVNT TIYPITSIPL
BplCESA4 477 HCRGWKSVYC MPKRPAFKGS APINLSDRLH QVLRWALGSV EIFLSRHCPL WYAYGG-KLK WLERMAYINT IVYPFTSIPL
BplCESA7 473 HCRGWRSIYC MPKRPAFKGT APINLSDRLN QVLRWALGSI EIFFSHHCPI WYGYKEGKLK WLERFSYVNT TVYPFTSLPL
BplCESA8 446 HCRGWRSVYC MPIRPAFKGS APINLSDRLH QVLRWALGSV EIFLSRHCPL WYGFAGGRLK WLQRLAYTNT IVYPFTSLPL
PpCESA8 476 HCRGWRSIYC MPHRPAFKGS APINLSDRLN QVLRWALGSV EISLSRHCPL WYGYG-R-LK CLERLAYINT TIYPLTSLPL
PtdCESA1 447 HCRGWRSIYC MPVRPAFKGS APINLSDRLH QVLRWALGSV EIFFSRHCPL WYGFGGGRLK WLQRLAYINT IVYPFTSLPL
PtdCESA2 474 HCRGWRSIYC MPKRAAFKGS APINLSDRLN QVLRWALGSV EIFFSGHSPN WYGYKKGKLK WLERFAYVNT TIYPFTSLAL
PtdCESA3 468 HCRGWRSVYC SPQRPAFKGS APINLSDRLH QVLRWALGSI EIFLSHHCPL WYGYGG-KLK LLERLAYINT IVYPFTSIPL
PtdCESA4 476 HARGWISIYC MPPRPAFKGS APINLSDRLN QVLRWALGSI EILLSRHCPI WYGYNGR-LK LLERLAYINT IVYPLTSLPL
PtdCESA5 457 HARGWRSIYC MPKRPAFKGS APINLSDRLN QVLRWALGSV EILLSRHCPI WYGYGGR-LK WLERFAYVNT TIYPITAIPL
PtdCESA6 476 HCHGWRSIYC IPSRPAFKGS APINLSDRLH QVLRWALGSV EIFLSRHCPL WYGYGGG-LK WLERLSYINA TVYPLTSIPL
PtdCESA7 476 HCHGWRSVYC IPKRPAFKGS APINLSDRLH QVLRWALGSV EIFFSRHCPI WYGYGGG-LK WLERFSYINS VVYPWTSIPL
PtiCESA1-A 476 HARGWISIYC MPPRPAFKGS APINLSDRLN QVLRWALGSI EILLSRHCPI WYGYSGR-LK LLERLAYINT IVYPLTSLPL
PtiCESA1-B 476 HARGWISIYC MPPRPAFKGS APINLSDRLN QVLRWALGSI EILLSRHCPI WYGYNGR-LK LLERLAYINT IVYPLTSLPL
PtiCESA3-A 453 HARGWRSIYC MPKRPAFKGS APINLSDRLN QVLRWALGSV EILLSRHCPI WYGYSGR-LK WLERFAYINT TIYPITAIPL
PtiCESA3-B 458 HARGWRSIYC MPKRPAFKGS APINLSDRLN QVLRWALGSV EILLSRHCPI WYGYSGR-LK WLERFAYINT TIYPITSIPL
PtiCESA3-C 457 HARGWRSIYC MPKRPAFKGS APINLSDRLN QVLRWALGSV EILLSRHCPI WYGYGGR-LK WLERFAYVNT TIYPITAIPL
PtiCESA3-D 457 HARGWRSIYC MPKRPAFKGS APINLSDRLN QVLRWALGSV EILLSRHCPI WYGYGGR-LK WLERFAYVNT TIYPITAIPL
PtiCESA4 468 HCRGWRSVYC SPQRPAFKGS APINLSDRLH QVLRWALGSI EIFLSHHCPL WYGYGG-KLK LLERLAYINT IVYPFTSIPL
PtiCESA6-A 476 HCHGWRSVYC IPKRPAFKGS APINLSDRLH QVLRWALGSV EIFFSRHCPI WYGYGGG-LK WLERFSYINS VVYPWTSIPL
PtiCESA6-B 476 HCHGWRSVYC IPKRPAFKGS APINLSDRLH QVLRWALGSV EIFFSRHCPI WYGYGGG-LK WLERFSYINS VVYPWTSIPL
PtiCESA6-C 476 HCHGWRSVYC TPKIPAFKGS APINLSDRLH QVLRWALGSV EILLSRHCPI WYGYGCG-LK WLERFSYINS VVYPLTSIPL
PtiCESA6-D 476 HCHGWRSVYC MPKRPAFKGS APINLSDRLH QVLRWALGSV EILLSRHCPI WYGYGCG-LK WLERFSYINS VVYPLTSIPL
PtiCESA6-E 476 HCHGWRSIYC IPSRPAFKGS APINLSDRLH QVLRWALGSV EIFLSRHCPL WYGYGGG-LK WLERLSYINA TVYPLTSIPL
PtiCESA6-F 476 HCHGWRSIYC IPARPAFKGS APINLSDRLH QVLRWALGSV EIFLSRHCPL WYGYGGG-LK WLERLSYINA TVYPLTSIPL
PtiCESA7-A 474 HCRGWRSIYC MPKLAAFKGS APINLSDRLN QVLRWALGSV EIFFSRHSPM LYGYKEGKLK WLERFAYVNT TIYPFTSLAL
PtiCESA7-B 474 HCRGWRSIYC MPKRAAFKGS APINLSDRLN QVLRWALGSV EIFFSRHSPM LYGYKEGKLK WLERFAYVNT TIYPFTSLAL
PtiCESA8-A 446 HCRGWRSIYC MPVRPAFKGS APINLSDRLH QVLRWALGSV EIFFSRHCPL WYGYGGGRLK WLQRLAYINT IVYPFTSLPL
PtiCESA8-B 446 HCRGWRSIYC MPVRPAFKGS APINLSDRLH QVLRWALGSV EIFFSRHCPL WYGFGGGRLK WLQRLAYINT IVYPFTSLPL

```
                    570        580        590        600        610        620        630        640
AtCESA1     555 IAYCILPAFC LITDRFIIPE ISNYASIWFI LLFISIAVTG ILELRWSGVS IEDWWRNEQF WVIGGTSAHL FAVFQGLLKV
AtCESA2     555 IVYCSLPAVC LLTGKFIVPE ISNYAGILFM LMFISIAVTG ILEMQWGGVG IDDWWRNEQF WVIGGASSHL FALFQGLLKV
AtCESA3     536 LMYCTLPAVC LFTNQFIIPQ ISNIASIWFL SLFLSIFATG ILEMRWSGVG IDEWWRNEQF WVIGGVSAHL FAVFQGILKV
AtCESA4     539 LAYCTIPAVC LLTGKFIIPT INNFASIWFL ALFLSIIATA ILELRWSGVS INDLWRNEQF WVIGGVSAHL FAVFQGLLKV
AtCESA5     555 LVYCSLPAIC LLTGKFIVPE ISNYASILFM ALFGSIAVTG ILEMQWGKVG IDDWWRNEQF WVIGGVSAHL FALFQGLLKV
AtCESA6     555 IVYCSLPAIC LLTGKFIVPE ISNYASILFM ALFSSIAITG ILEMQWGKVG IDDWWRNEQF WVIGGVSAHL FALFQGLLKV
AtCESA7     554 LAYCTLPAIC LLTDKFIMPP ISTFASLFFI SLFMSIIVTG ILELRWSGVS IEEWWRNEQF WVIGGTSAHL FAVVQGLLKI
AtCESA8     526 VAYCTLPAIC LLTGKFIIPT LSNLASMLFL GLFISIILTS VLELRWSGVS IEDLWRNEQF WVIGGVSAHL FAVFQGFLKM
AtCESA9     555 LVYCSLPAIC LLTGKFIVPE ISNYAGILFL LMFMSIAVTG ILEMQWGKIG IDDWWRNEQF WVIGGVSSHL FALFQGLLKV
AtCESA10    549 LAYCMLPAFC LITNTFIIPE ISNLASLCFM LLFASIYASA ILELKWSDVA LEDWWRNEQF WVIGGTSAHL FAVFQGLLKV
BplCESA3    536 LMYCTLPAVC LLTNKFIIPQ ISNVASIWFI SLFLSIFATG ILEMRWSGVG IDEWWRNEQF WVIGGVSAHL FAVCQGLLKV
BplCESA4    556 LAYCTLPAVC LLTGKFIIPT LTNLASIYFM ALFLSIIATG VLELRWSGVS IEDLWRNEQF WVIGGVSAHL FAVFQGLLKV
BplCESA7    553 LAYCTLPAIC LLTDKFIMPP ISTFASLYFI ALFMSIFITG ILELRWSGVT IEEWWRNEQF WVIGGVSAHL FAVFQGLLKV
BplCESA8    526 VAYCVIPAIC LLTGKFIIPT LSNLASMLFL GLFISIIVTS VLELRWSGVS IEEWWRNEQF WVIGGVSAHL FAVFQGFLKM
PpCESA8     554 VAYCTLPAVC LLTGKFIIPT ISNLDSLWFI SLFMSIFITG ILEMRWSGVG IDEWWRNEQF WVIGGVSAHL FALFQGLLKV
PtdCESA1    527 IAYCTIPAVC LLTGKFIIPT LSNLASMLFL GLFISIIVTA VLELRWSGVS IEDLWRNEQF WVIGGVSAHL FAVFQGFLKM
PtdCESA2    554 VAYCCLPAIC LLTDKFIMPE ISTFASLFFI ALFLSIFSTG ILELRWSGVS IEEWWRNEQF WVIGGVSAHL FAVVQGLLKV
PtdCESA3    547 LAYCTIPAVC LLTGKFIIPT LNNLASIWFL GPFHLNHSNI CVGTSWSGVS IQDLWRNEQF WVIGGVSAHL FAVFQGLLKV
PtdCESA4    555 LAYCVLPAVC LVSGKFIIPE ISNYASMWFI LLFISIFATG ILELRWSGVG IEDWWRNEQF WVIGGTSAHL FAVFQGLLKV
PtdCESA5    536 LMYCTLPAIC LLTDKFIIPQ ISNIASIWFI SLFLSIFATG ILEMRWSGVG IDEWWRNEQF WVIGGVSAHL FAVFQGLLKV
PtdCESA6    555 LAYCTLPAVC LLTGKFITPE LSNAASLWFL SLFICIFATS ILEMRWSGVG IDEWWRNEQF WVIGGVSAHL FAVFQGLLKV
PtdCESA7    555 LIYCTLPAIC LLTGKFIVPE ISNYASIVFI ALFISIAATG ILEMQWGGVG IDDWWRNEQF WVIGGVSSHL FALFQGLLKV
PtiCESA1-A  555 LAYCILPAIC LVTGKFIIPE ISNYAGMWFI LLFISIFATG ILELRWSGVG IEDWWRNEQF WVIGGTSAHL FAVFQGLLKV
PtiCESA1-B  555 LAYCVLPAVC LIS------- --NYASMWFI LLFISIFATG ILELRWSGVG IEDWWRNEQF WVIGGTSAHL FAVFQGLLKV
PtiCESA3-A  532 LAYCTLPAVC LLTGKFIIPQ ISNIASIWFI SLFLSIFATG ILEMRWSGVG IDEWWRNEQF WVIGGVSAHL FAVFQGLLKV
PtiCESA3-B  537 LAYCTLPAVC LLTGKFIIPQ ISNIASIWFI SLFLSIFATG ILEMRWSGVG IDEWWRNEQF WVIGGVSAHL FAVFQGLLKV
PtiCESA3-C  536 LFYCTLPAIC LLTDKFIIPQ ISNIASIWFI SLFLSIFATG ILEMRWSGVG IDEWWRNEQF WVIGGVSAHL FAVFQGLLKV
PtiCESA3-D  536 LLYCTLPAIC LLTDKFIIPQ ISNIASIWFI SLFLSIFATG ILEMRWSGVG IDEWWRNEQF WVIGGVSAHL FAVFQGLLKV
PtiCESA4    547 LAYCTIPAVC LLTGKFIIPT LNNLASIWFL ALFLSIIATS VLELRWSGVS IQDLWRNEQF WVIGGVSAHL FAVFQGLLKV
PtiCESA6-A  555 LVYCTLPAIC LLTGKFIVPE ISNYASIVFI ALFISIAATG ILEMQWGGVG IDDWWRNEQF WVIGGVSSHL FALFQGLLKV
PtiCESA6-B  555 LVYCTLPAIC LLTGKFIVPE ISNYASIVFM ALFISIAATG ILEMQWGGVG IHDWWRNEQF WVIGGASAHL FALFQGLLKV
PtiCESA6-C  555 IAYCTLPAVC LLTGKFIVPE ISNYASIIFM ALFISIAATG ILEMQWGGVG IHDWWRNEQF WVIGGASSHL FALFQGLLKV
PtiCESA6-D  555 IAYCTLPAVC LLTGKFIVPE ISNYASIIFM ALFISIAATG ILEMQWGGVG IHDWWRNEQF WVIGGASAHL FALFQGLLKV
PtiCESA6-E  555 LAYCTLPAVC LLTGKFITPE LSNAASLWFL SLFICIFATS ILEMRWSGVG IDEWWRNEQF WVIGGVSAHL FAVFQGLLKV
PtiCESA6-F  555 LAYCTLPAVC LLTGKFITPE LSNAASLWFL SLFICIFATS ILEMRWSGVG IDEWWRNEQF WVIGGVSAHL FAVFQGLLKV
PtiCESA7-A  554 VAYCCLPAIC LLTDKFIMPE ISTFASLFFI GLFLSIFSTG ILELRWSGVS IEEWWRNEQF WVIGGVSAHL FAVVQGLLKV
PtiCESA7-B  554 VAYCCLPAIC LLTDKFIMPE ISTFASLFFI GLFLSIFSTG ILELRWSGVS IEEWWRNEQF WVIGGVSAHL FAVVQGLLKV
PtiCESA8-A  526 IAYCTIPAVC LLTGKFIIPT LSNLASMLFL GLFISIIVTA VLELRWSGVS IEDLWRNEQF WVIGGVSAHL FAVFQGFLKL
PtiCESA8-B  526 IAYCTIPAVC LLTGKFIIPT LSNLASMLFL GLFISIIGTA VLELRWSGVS IEDLWRNEQF WVIGGVSAHL FAVFQGFLKM
```

```
                    650        660        670        680        690        700        710        720
AtCESA1     635 LAGIDTNFTV TSK-ATDEDG DFAELYIFKW TALLIPPTTV LLVNLIGIVA GVSYAVNSGY QSWGPLFGKL FFALWVIAHL
AtCESA2     635 LAGVNTNFTV TSK-AAD-DG AFSELYIFKW TTLLIPPTTL LIINIIGVIV GVSDAISNGY DSWGPLFGRL FFALWVIVHL
AtCESA3     616 LAGIDTNFTV TSK-ASDEDG DFAELYLFKW TTLLIPPTTL LIVNLVGVVA GVSYAINSGY QSWGPLFGKL FFAFWVIVHL
AtCESA4     619 LFGVDTNFTV TSKGASDEAD EFGDLYLFKW TTLLIPPTTL IILNMVGVVA GVSDAINNGY GSWGPLFGKL FFAFWVIVHL
AtCESA5     635 LAGVETNFTV TSK-AAD-DG EFSELYIFKW TSLLIPPTTL LIINVIGVIV GISDAISNGY DSWGPLFGRL FFAFWVILHL
AtCESA6     635 LAGVDTNFTV TSK-AAD-DG EFSDLYLFKW TSLLIPPMTL LIINVIGVIV GVSDAISNGY DSWGPLFGRL FFALWVIIHL
AtCESA7     634 LAGIDTNFTV TSK-ATD-DD DFGELYAFKW TTLLIPPTTV LIINIVGVVA GISDAINNGY QSWGPLFGKL FFSFWVIVHL
AtCESA8     606 LAGLDTNFTV TSK--TADDL EFGELYIVKW TTLLIPPTSL LIINLVGVVA GFSDALNKGY EAWGPLFGKV FFAFWVILHL
AtCESA9     635 LAGVSTNFTV TSK-AAD-DG EFSELYLFKW TSLLIPPTTL LIINVIGVIV GVSDAINNGY DSWGPLFGRL FFALWVIVHL
AtCESA10    629 FAGIDTNFTV TSK-ASDEDG DFAELYVFKW TSLLIPPTTI LVVNLVGIVA GVSYAINSGY QSWGPLMGKL LFAFWVVAHL
BplCESA3    616 LAGIDTNFTV TSK-ASDEDG DFTELYMFKW TTLLIPPTTL LIINLVGVVA GISYAINSGY QSWGPLFGKL FFAFWVIVHL
BplCESA4    636 LAGVDTNFTV TSK--TADDA EFGELYMFKW TTLLIPPTTL IILNMVGVVA GVSDAINNGY GSWGPLFGKL FFAFWVIVHL
BplCESA7    633 LAGIDTNFTV TSK-ATD-DE DFGELYTFKW TTLLIPPTTI LIINLVGVVA GISDAINNGY ESWGPLFGKL FFAFWVIVHL
BplCESA8    606 LAGVDTNFTV TTK--AADDA EFGELYIIKW TTVLIPPTTL IIVNMVGVVA GFSDALNKGY EAWGPLFGKV FFAFWVILHL
PpCESA8     634 FAGIDTNFTV TSK-TGE-DE DFGELYTLKW TSLLIPPTTL LLFNMVGVVA GISDAINNGY SAWGPLFGKL FFAFWVIVHL
PtdCESA1    607 LAGIDTNFTV TAK--AAEDA EFGELYMVKW TTLLIPPTTL LIINMSG-CA GFSDALNKGY EAWGPLFGKV FFAFWVILHL
PtdCESA2    634 LAGIDLNFTV TSK-ATD-DD DFGELYAFKW TTLLIPPTTI LIINLVGVVA GVSDAINNGY QSWGPLFGKL FFAFWVIVHL
PtdCESA3    627 LAGVDTNFTV TSK--SADDA EFGELYLFKW TTLLIPPTTL IILNMVGVVA GVSDAINNGY GSWGPLFGKL FFAFWVIVHL
PtdCESA4    635 LAGIDTNFTV TSK-ASDEDG DFAELYVFKW TSLLIPPTTV IVLNMVGIVA GVSYAINSGY QSWGPLFGKL FFAIWVIAHL
PtdCESA5    616 LAGIDTNFTV TSK-ASDEDG DSAELYLFKW TTLLIPPTTL LIVNLVGVVA GISHAINSGY QSWGPLFGKL FFAFWVIVHL
PtdCESA6    635 LAGVDTNFTV TSK-GGD-DD EFSELYAFKW TTLLIPPTTL LIINLVGVVA GVSNAINNGY ESWGPLFGKL FFAFWVIVHL
PtdCESA7    635 LAGVSTNFTV TSK-GAD-DG EFSELYLFKW TSLLIPPTTL LIMNLVGVVV GVSDAINNGY DSWGPLFGRL FFALWVILHL
PtiCESA1-A  635 LAGIDTNFTV TSK-ASDEDG DFAELYVFKW TSLLIPPTTV ILLNMMGIVA GVSFAINSGY QSWGPLFGKL FFAIWVIAHL
PtiCESA1-B  626 LAGIDTNFTV TSK-ASDEDG DFAELYVFKW TSLLIPPTTV ILLNMVGIVA GVSYAINSGY QSWGPLFGKL FFAIWVIAHL
PtiCESA3-A  612 LAGIDTNFTV TSK-ASDEDG DFTELYMFKW TTLLIPPTTL LIINLVGVVA GVSYAINSGY QSWGPLFGKL FFAFWVIIHL
PtiCESA3-B  617 LAGIDTNFTV TSK-ASDEDG DFTELYMFKW TTLLIPPTTL LIINLVGVVA GVSYAINSGY QSWGPLFGKL FFAFWVIIHL
PtiCESA3-C  616 LAGIDTNFTV TSK-SSDEDG DFTELYMFKW TTLLIPPTTL LIVNLVGVVA GISHAINSGY QSWGPLFGKL FFAFWVIVHL
PtiCESA3-D  616 LAGIDTNFTV TSK-ASDEDG GFAELYLFKW TTLLIPPTTL LIVNLVGVVA GISHAINSGY QSWGPLFGKL FFAFWVIVHL
PtiCESA4    627 LGGVDTNFTV TSK--SADDA EFGELYLFKW TTLLIPPTTL IILNMVGVVA GVSDAINNGY GSWGPLFGKL FFAFWVIVHL
PtiCESA6-A  635 LAGVSTNFTV TSK-GAD-DG EFSELYIFKW TSLLIPPTTL LIMNIVGVVV GVSDAINNGY DSWGPLFGRL FFALWVILHL
PtiCESA6-B  635 LAGVSTNFTV TSK-AAD-DG EFSELYLFKW TSLLIPPTTL LIMNIVGVVV GVSDAINNGY DSWGPLFGRL FFALWVILHL
PtiCESA6-C  635 LAGVNTNFTV TSK-AAD-DG EFSELYLFKW TSLLIPPMTL LIINIIGVVV GISDAINNGY ETWGPLFGKL FFALWVIVHL
PtiCESA6-D  635 LAGVNTNFTV TSK-AAD-DG EFSDLYLFKW TSLLIPPMTL LIINIIGVVV GISDAINNGY ETWGPLFGKL FFALWVIVHL
PtiCESA6-E  635 LAGVDTNFTV TSK-GGD-DD EFSELYAFKW TTLLIPPTTL LIINLVGVVA GVSNAINNGY ESWGPLFGKL FFAFWVIVHL
PtiCESA6-F  635 LAGVDTNFTV TSK-GGD-DD EFSELYAFKW TTLLIPPTTL LIINLVGVVA GVSNAINNGY ESWGPLFGKL FFAFWVIVHL
PtiCESA7-A  634 LAGIDTNFTV TSK-ATD-DD DFGELYAFKW TTLLIPPTTI LIINLVGVVA GVSDAINNGY QSWGPLFGKL FFAFWVIVHL
PtiCESA7-B  634 LAGIDTNFTV TSK-ATD-DD DFGELYAFKW TTLLIPPTTI LIINLVGVVA GVSDAINNGY QSWGPLFGKL FFAFWVIVHL
PtiCESA8-A  606 LAGIDTNFTV TAK--AADDT EFGELYMVKW TTLLIPPTTL LIINLVGVVA GFSDALNKGY EAWGPLFGKV FFALWVILHL
PtiCESA8-B  606 LAGIDTNFTV TAK--AAEDT EFGELYMVKW TTLLIPPTTL LIINLVGVVA GFSDALNKGY EAWGPLFGKV FFAFWVILHL
```

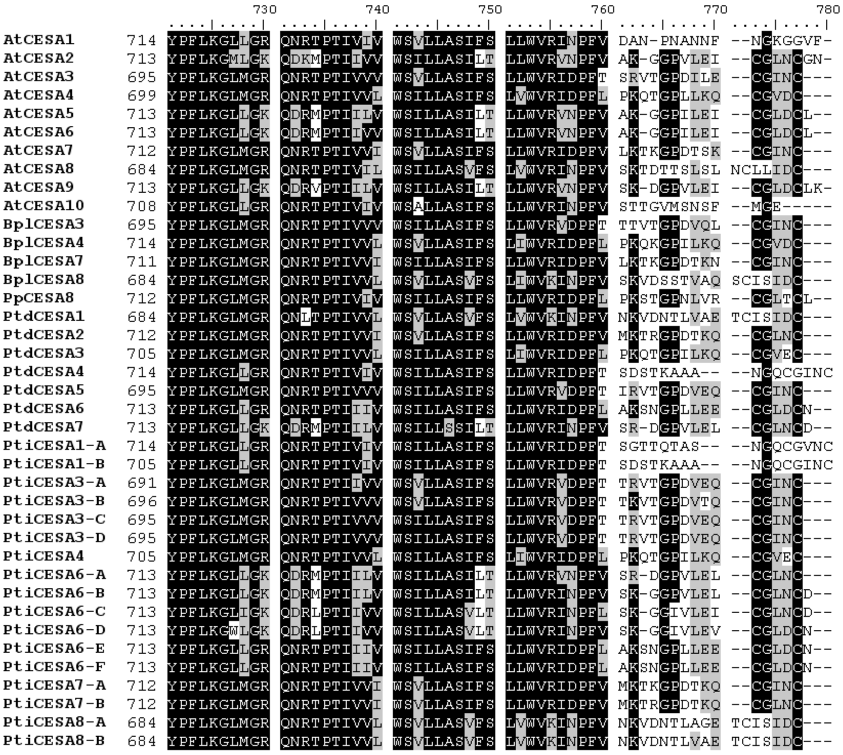

Figure S2. Gel electrophoresis result of *Bplactin* and *BplCesAs* RT-PCR products from different birch tissues. **a** showing the stable trancript expression abundance of *Bplactin* in leaves (Lanes 1–5) and stem (Lanes 6–10). **b** showing the specific amplification of four *BplCesAs* (in the order of *BplCesA1*, *BplCesA2*, *BplCesA3* and *BplCesA4*) in leaf (1–4) and stem (5–8) by specific primers.

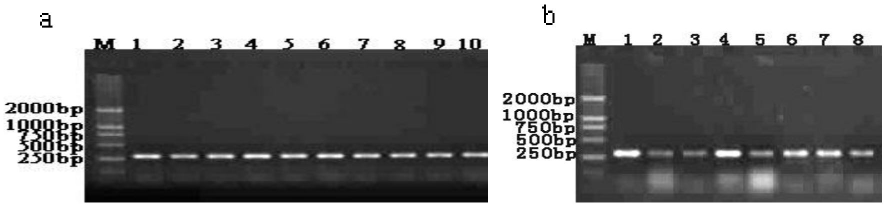

Supplement: Supplementary file 1 [file ijms-13-12195-s001.pdf]
